# Effects of circadian clock disruption on gene expression and biological processes in* Aedes aegypti*

**DOI:** 10.1186/s12864-024-10078-8

**Published:** 2024-02-13

**Authors:** Vinaya Shetty, Zach N. Adelman, Michel A. Slotman

**Affiliations:** Department of Entomology, Texas A&M University, College station, TX 77843 USA

**Keywords:** *Aedes aegypti*, Circadian clock, Gene expression, Metabolic processes, Transcriptome, Rhythmic genes, Mosquitoes

## Abstract

**Background:**

This study explores the impact of disrupting the circadian clock through a *Cycle* gene knockout (KO) on the transcriptome of *Aedes aegypti* mosquitoes. The investigation aims to uncover the resulting alterations in gene expression patterns and physiological processes.

**Results:**

Transcriptome analysis was conducted on *Cyc* knockout (*AeCyc*^-/-^) and wild-type mosquitoes at four time points in a light-dark cycle. The study identified system-driven genes that exhibit rhythmic expression independently of the core clock machinery. *Cyc* disruption led to altered expression of essential clock genes, affecting metabolic processes, signaling pathways, stimulus responses and immune responses. Notably, gene ontology enrichment of odorant binding proteins, indicating the clock's role in sensory perception. The absence of *Cyc* also impacted various regulation of metabolic and cell cycle processes was observed in all time points.

**Conclusions:**

The intricate circadian regulation in *Ae. aegypti* encompasses both core clock-driven and system-driven genes. The KO of *Cyc* gene instigated extensive gene expression changes, impacting various processes, thereby potentially affecting cellular and metabolic functions, immune responses, and sensory perception. The circadian clock's multifaceted involvement in diverse biological processes, along with its role in the mosquito's daily rhythms, forms a nexus that influences the vector's capacity to transmit diseases. These insights shed light on the circadian clock's role in shaping mosquito biology and behavior, opening new avenues for innovative disease control strategies.

**Supplementary Information:**

The online version contains supplementary material available at 10.1186/s12864-024-10078-8.

## Background

The circadian clock is a conserved biological mechanism found in various organisms, including insects, that regulates the timing of physiological and behavioral processes [1]. The clock enables organisms to anticipate and adapt to cyclic environmental changes, thereby playing a crucial role in their survival and fitness. In insects, the circadian clock governs essential aspects of their biology, including feeding behavior, flight activity, mating, and vector competence [2]. Different species of mosquitoes exhibit distinct temporal patterns in their feeding behaviors, with some being predominantly active during the evening or night, while others are more active during the early morning or daytime [3].

The mosquito *Aedes aegypti* is the primary vector responsible for transmitting several arboviruses, including dengue, Zika, and chikungunya viruses [4, 5], displays specific temporal coordination of behaviors such as host-seeking, blood-feeding, and oviposition, which are tightly regulated by the circadian clock [6]. Studies have shown that *Aedes aegypti* mosquitoes are predominantly day-biting, with peak activity during the early morning and late afternoon [7]. The temporal synchronization of mosquito feeding patterns with the host's activity influences the efficiency of pathogen transmission. Perturbations in the circadian system could disrupt these behaviors and subsequently impact the transmission dynamics of mosquito-borne diseases [8]. Understanding the relevance of circadian rhythms to the temporal dynamics of mosquito species is crucial for comprehending their vector competence. Disruptions in the circadian clock may alter the timing of mosquito biting, affecting the transmission efficiency of pathogens due to mismatches in host availability and mosquito activity. Moreover, some mosquito species, such as Anopheles mosquitoes, which are vectors for malaria parasites, exhibit nocturnal feeding patterns [9, 10], further emphasizing the importance of circadian regulation in vector biology. In a previous study, we explored the consequences of disabling the circadian clock in *Ae. aegypti*, revealing significant effects on the mosquito's reproductive fitness, longevity, and feeding behavior [11]. Building upon these findings, our objective in this study was to investigate the impact of circadian clock disruption on gene expression profiles and underlying biological processes in *Ae. aegypti*. Thus, to achieve this, we performed RNA-seq on a *Cyc*^*-/-*^* Ae. aegypti* line (*AeCyc*^*-/-*^), lacking a functional circadian clock due to a mutation in the core clock gene. By comparing the transcriptomic profiles of *Cyc*^*-/-*^ mosquitoes with wild-type (WT) individuals under a light-dark (LD) cycle, we elucidated the intricate network of genes and pathways influenced by the absence of a functional circadian clock. Understanding the molecular mechanisms governing their circadian rhythm and related physiological processes may offer valuable insights into regulating mosquito biology, vector competence, and contribute to innovative strategies for controlling mosquito populations and curtailing mosquito-borne diseases.

## Results

### Transcriptome profiling and rhythmic differential gene expression analysis

**Fig. 1:**
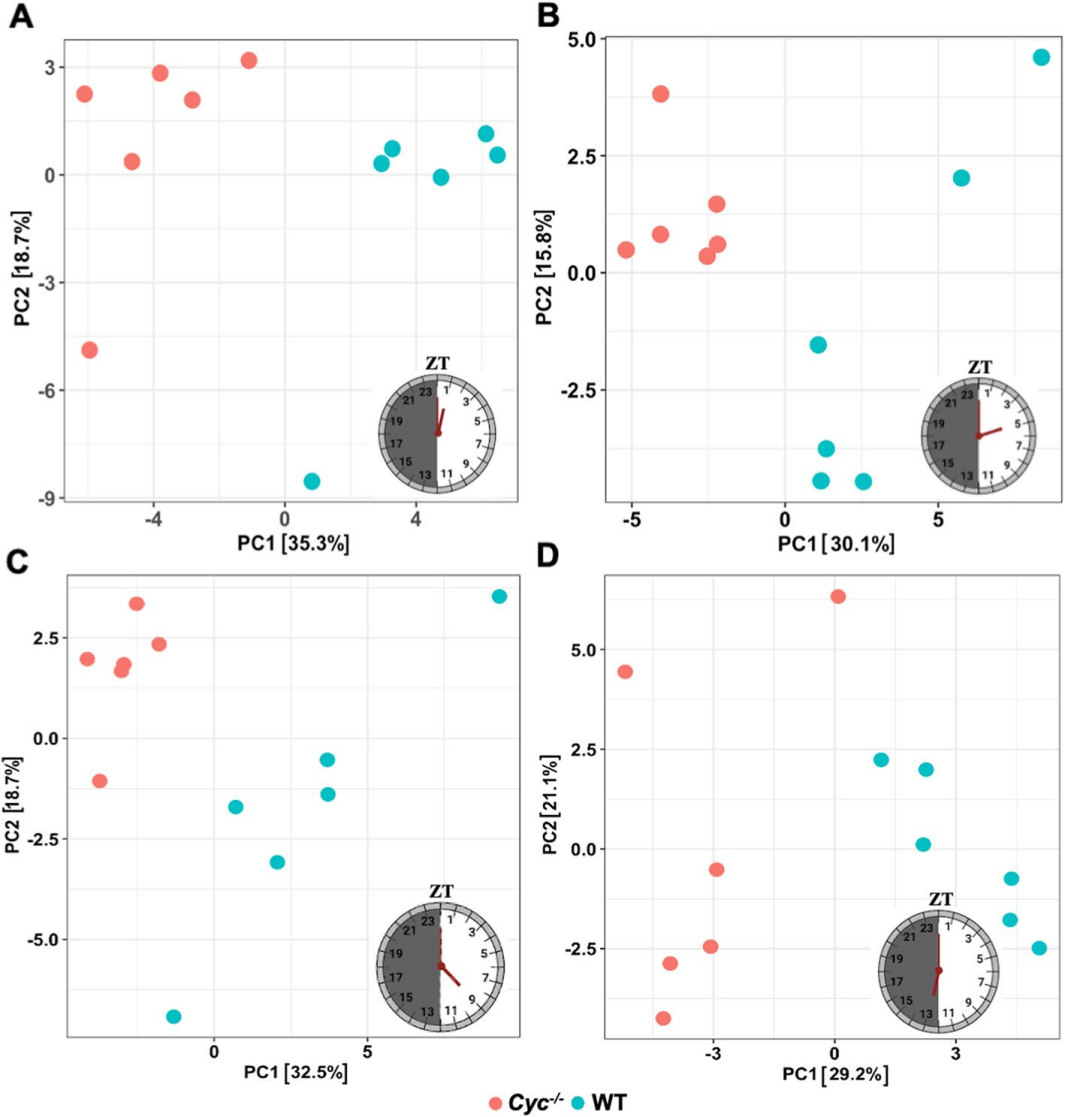
Principal component analysis (PCA) for WT and *AeCyc*^*-/-*^ at four different time points in a LD cycle (**A**) ZT1, (**B**) ZT5, (**C**) ZT9 and (**D**) ZT13

In this study, we conducted a transcriptomic analysis of the *Cyc*^-/-^ *Aedes aegypti* line and the wild-type control at four different time points in a LD cycle, namely ZT1 (7 AM), ZT5 (11 AM), ZT9 (3 PM), and ZT13 (7 PM), with six replicates at each time point, resulting in a total of 48 samples analyzed. We generated more than 503 millon 100-bp cDNA reads across eight experimental groups, successfully mapping over 94% of the reads to the *Ae. aegypti* genome (version: AaegL5_2) from Vectorbase (Supplementary file 1: Table S1). To visualize the level of variation in gene expression across different samples, we performed a principal component analysis (PCA) using the normalized gene counts of each sample at different time points in a LD cycle (Fig. 1). The first component (PC1) accounted for 35.3% (ZT1), 30.1% (ZT5), 32.5% (ZT9), and 29.2% (ZT13) of the variance in gene expression, effectively separating *Cyc*^*-/-*^ from WT *Ae. aegypti* samples. The second component (PC2) accounted for 18.7% (ZT1), 15.8% (ZT5), 18.7% (ZT9), and 21.1% (ZT13) of the variance and reflected differences between *Cyc*^*-/-*^ and WT samples.

**Fig. 2:**
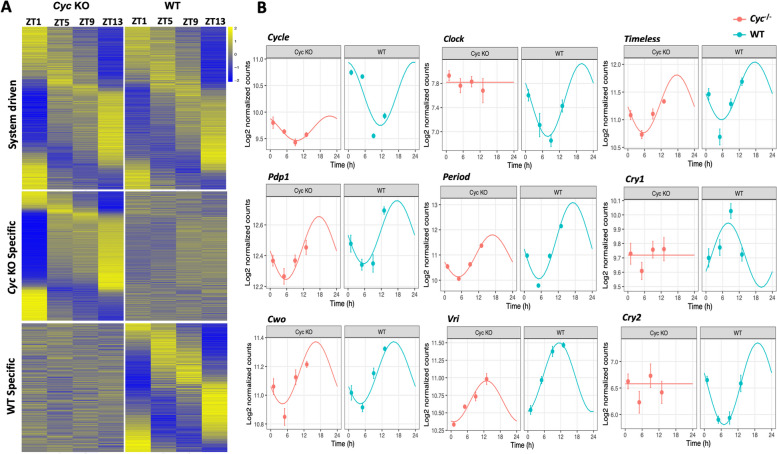
Comparing the rhythmic gene expression patterns between WT and *AeCyc*^*-/-*^*.* (**A**) Heat maps of normalized rhythmic mRNA levels for genes in the *Cyc* KO and WT *Ae. aegypti.* Genes were classified as system driven, *Cyc* KO specific and WT specific. (**B**) Temporal expression pattern of circadian clock genes

To explore genes expressed rhythmically in the whole body that may regulate physiological and behavioral rhythms in *Ae. aegypti*, we tested goodness of fit using *dryR* and found overall good fits for phase, mean, and amplitude. To further distinguish the specific contribution of the core clock oscillator on rhythmic gene expression in *Ae. aegypti* and its impact on physiological function, we focused our analysis on comparing differential rhythmic expression between *Cyc* KO and WT in the LD cycle. Using *dryR*, genes were grouped based on their temporal expression patterns into three categories: system-driven, *Cyc* KO-specific, and WT-specific (Fig. 2A). Among all rhythmic genes in WT *Ae. aegypti*, 49.5% were independent of a functional clock and remained rhythmic in *Cyc* KO, named as system driven (Supplementary file 2: S5-S5.5). These genes exhibited lower amplitudes compared to clock-driven or clock-modulated genes and showed a bimodal phase distribution. Our analysis revealed that system-driven rhythmic genes are significantly enriched in several important physiological pathways. Notably, the pathways implicated include Glycolysis/Gluconeogenesis, Oxidative phosphorylation, Wnt signaling pathway, Ubiquitin-mediated proteolysis, Citrate cycle (TCA cycle) etc., These pathways constitute a foundation for further exploration of the regulatory roles of system-driven rhythmic genes in fundamental cellular processes, highlighting potential implications for physiological conditions and daily activity.

On the other hand, 33.9% were *Cyc* KO-specific and 16.4% were WT-specific. Analyzing differential rhythmicity between rhythmic gene expression in wild-type and *Cyc* KO revealed that the expression of most rhythmic genes in wild-type *Ae. aegypti* was affected in *Cyc* KO, with this effect being significantly higher than background (calculated using genes arrhythmically expressed in wild type). Consequently, only genes with an adjusted *p*-value cutoff of ≤ 0.05 were considered in subsequent analysis. In addition to that, we then analyzed the impact of *Cyc* KO on the relative expression and rhythmic appearance of nine essential circadian clock genes: *Cyc (Cycle), Clk (Clock), Per (Period), Tim (Timeless)*, *Cry1 (Cryptochrome-1), Cry2* (*Cryptochrome-2), Pdp1(Par domain protein 1*)*, Vri (Vrille)*, and *Cwo (Clockworkorange)* at 5-hour intervals in light:dark conditions between ZT1 to ZT13 (Fig. 2B). The results revealed that some circadian clock genes lost rhythmicity in the absence of *Cyc*. Surprisingly, *AeCyc*^*−/−*^ mRNA was still detected, albeit at significantly lower levels compared to WT. *Clk, Cry1,* and *Cry2* were completely arrhythmic and significantly higher at some time points, in antiphase pattern compared to WT in LD conditions. In contrast, *Per* and *Vri* gene transcripts did not show significant differences compared to WT during the light phase. However, at ZT13, they exhibited significantly lower expression in *AeCyc*^*−/−*^ compared to WT, where they peak early in the night. Conversely, *Tim*, *Pdp1*, and *Cwo* did not display significant differences in expression levels compared to WT.

### Differentially gene expression of *Cyc*^-/-^ and WT

**Fig. 3:**
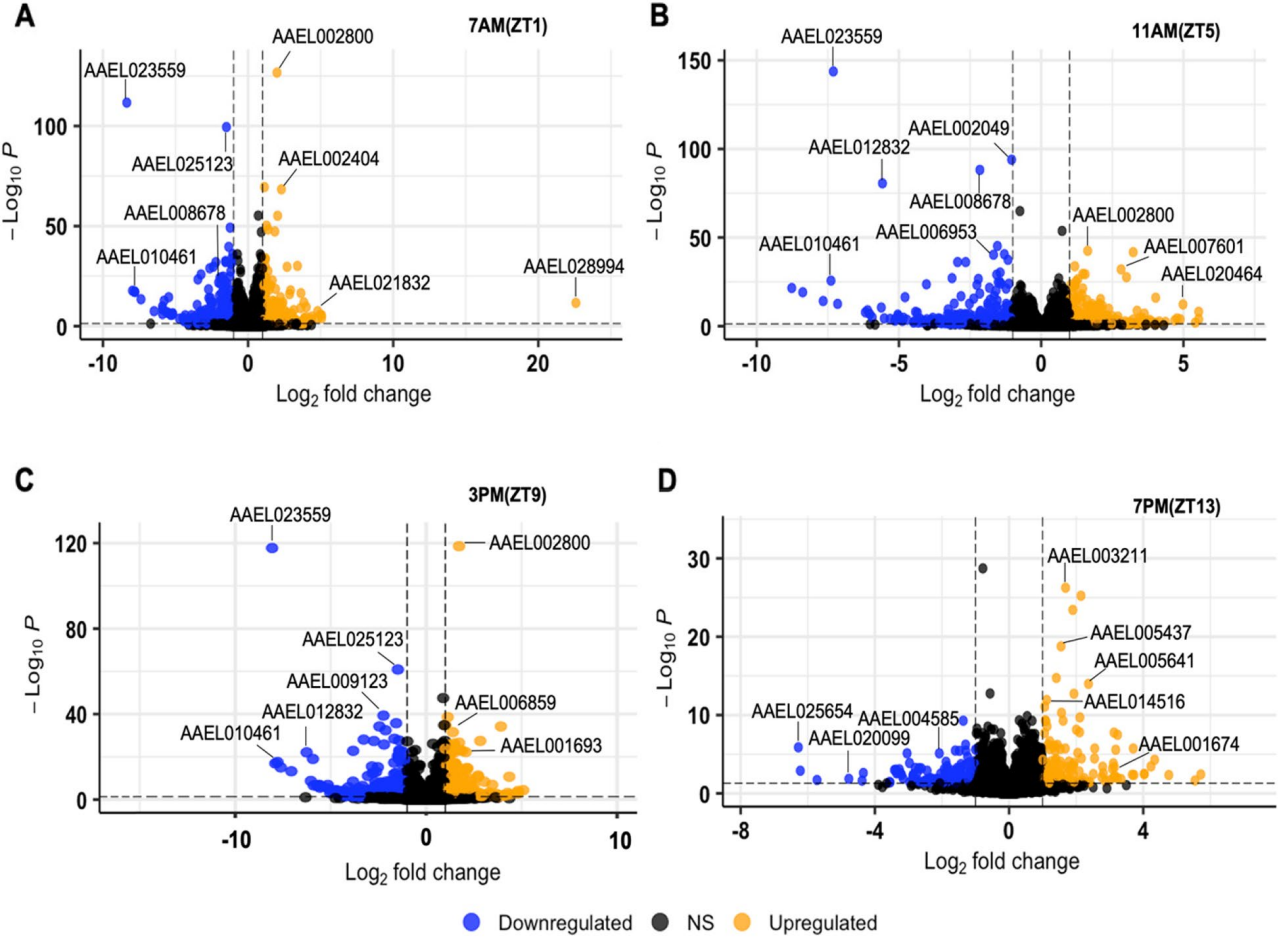
Volcano plots for differentially expressed up and down regulated genes at four different time points in a LD cycle (**A**) ZT1, (**B**) ZT5, (**C**) ZT9 and (**D**) ZT13. Dotted horizontal lines indicate *p*-value thresholds. Dotted vertical lines indicate ± 2-fold change. Blue dots represent significantly down-regulated genes; orange dots represent significantly up-regulated genes

Next, we identified transcripts significantly differentially expressed [log2(Fold Change) > 1, Benjamini-Hochberg adjusted *p*-value < 0.05 from a Wald test] in *AeCyc*^-/-^ compared to the WT at each time point (Fig. 3). Our analysis revealed 5764 (ZT1), 4099 (ZT5), 4382 (ZT9), and 2658 (ZT13) genes to be differentially expressed in *AeCyc*^-/-^ compared to WT *Ae. aegypti*.

The volcano plots generated for four different time points in a LD cycle (Fig. 3) revealed differentially expressed genes (DEGs) with both up and downregulation (padj<0.05, log2FC≥1). The DEGs recorded in the volcano plot and Pfam database was used to identify protein domain names (Supplementary file 1: Table S2). The highest number of upregulated genes was observed at ZT13 in the absence of light. The gene AAEL002800 (DNA polymerase epsilon) was upregulated in all three time points except at ZT13. Additionally, several genes, such as AAEL003211, AAEL005437, and AAEL005641, were significantly upregulated at ZT13. Regarding downregulated genes, the top ones were AAEL023559 (Peptidase M2), AAEL025123 (GRIP domain), and AAEL010461 (Peroxisome membrane protein, Pex16) at all three time points (ZT1, ZT5 and ZT9) in the presence of light. Gene AAEL008678 (Calponin homology domain) was downregulated at ZT1 and ZT5, and AAEL012832 (Cytochrome b561) was downregulated at ZT5 and ZT9. No similar downregulated genes were found at ZT13.

**Fig. 4:**
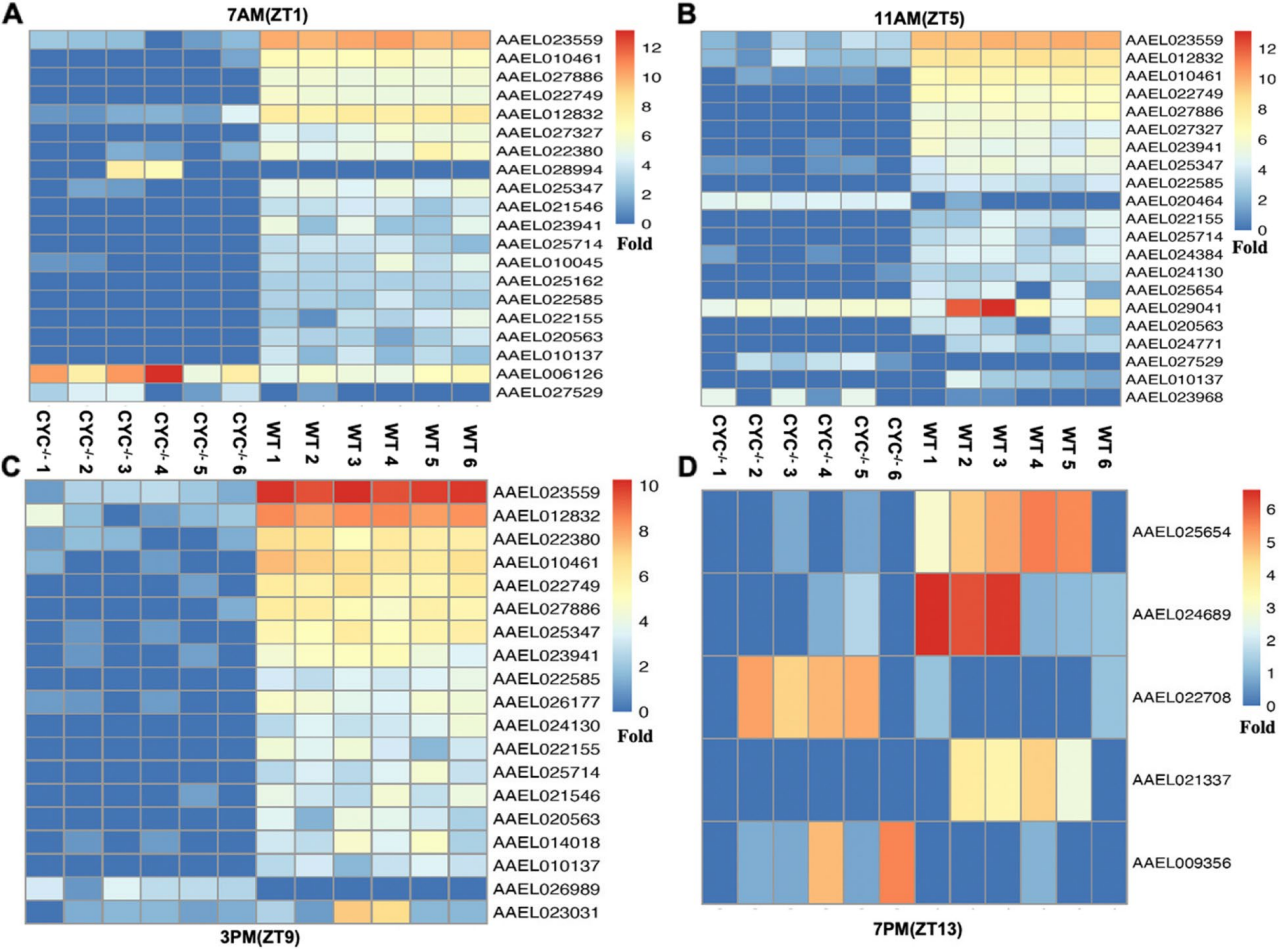
Heat maps showing differentially expressed genes (>5-fold changes) between WT and *AeCyc*^*-/-*^ at four different time points in a LD cycle (**A**) ZT1, (**B**) ZT5, (**C**) ZT9 and (**D**) ZT13

A heatmap was constructed using DEGs with more than five-fold (>5) differences (Fig. 4, Supplementary file 1: Table S3). The heatmap demonstrated significant downregulation of a large number of DEGs at ZT1, ZT5, and ZT9, with very few showing >5-fold differential expression at ZT13. Specific genes, such as AAEL023559 (Angiotensin-converting enzyme/Peptidase M2), AAEL012832 (Cytochrome B561), AAEL010461 (Peroxisomal membrane protein, PEX16), AAEL027327 (stress response protein NST1-like), and AAEL029041 (Cecropin-B type 2), were differentially expressed at different time points. Overall, these results indicate that presence of light significantly affects the differential expression of genes in *AeCyc*^-/-^ compared to WT.

### GO enrichment analysis

We conducted GO enrichment analyses separately for *AeCyc*^-/-^ and WT specific rhythmic genes and, also, up and down regulation of DEGs. The GO analysis revealed that rhythmic genes in the *Ae. aegypti cycle* knockout belong to diverse biological processes, including DNA topological change, several metabolic processes, and protein phosphorylation (Supplementary file 1: Figure S1A, Supplementary file 2: S5.3). KEGG pathway enrichment analysis further revealed that the Fanconi anemia pathway, MAPK signaling pathway, and ubiquitin-mediated proteolysis were among the most enriched rhythmic pathways in the *Cyc*^-/-^ specific *Ae. aegypti* (Supplementary file 1: Figure S1B). On the other hand, GO enrichment analysis for WT specific rhythmically expressed genes (Supplementary file 1: Figure S1C, Supplementary file 2: S5.4) showed biological processes related to carboxylic acid biosynthetic process, fatty acid metabolic process, lipid metabolic process, etc. Further, KEGG pathway analysis for WT specific rhythmic genes revealed enrichment in fatty acid biosynthesis, longevity regulating pathway, and metabolic pathways (Supplementary file 1: Figure S1D).

**Fig. 5:**
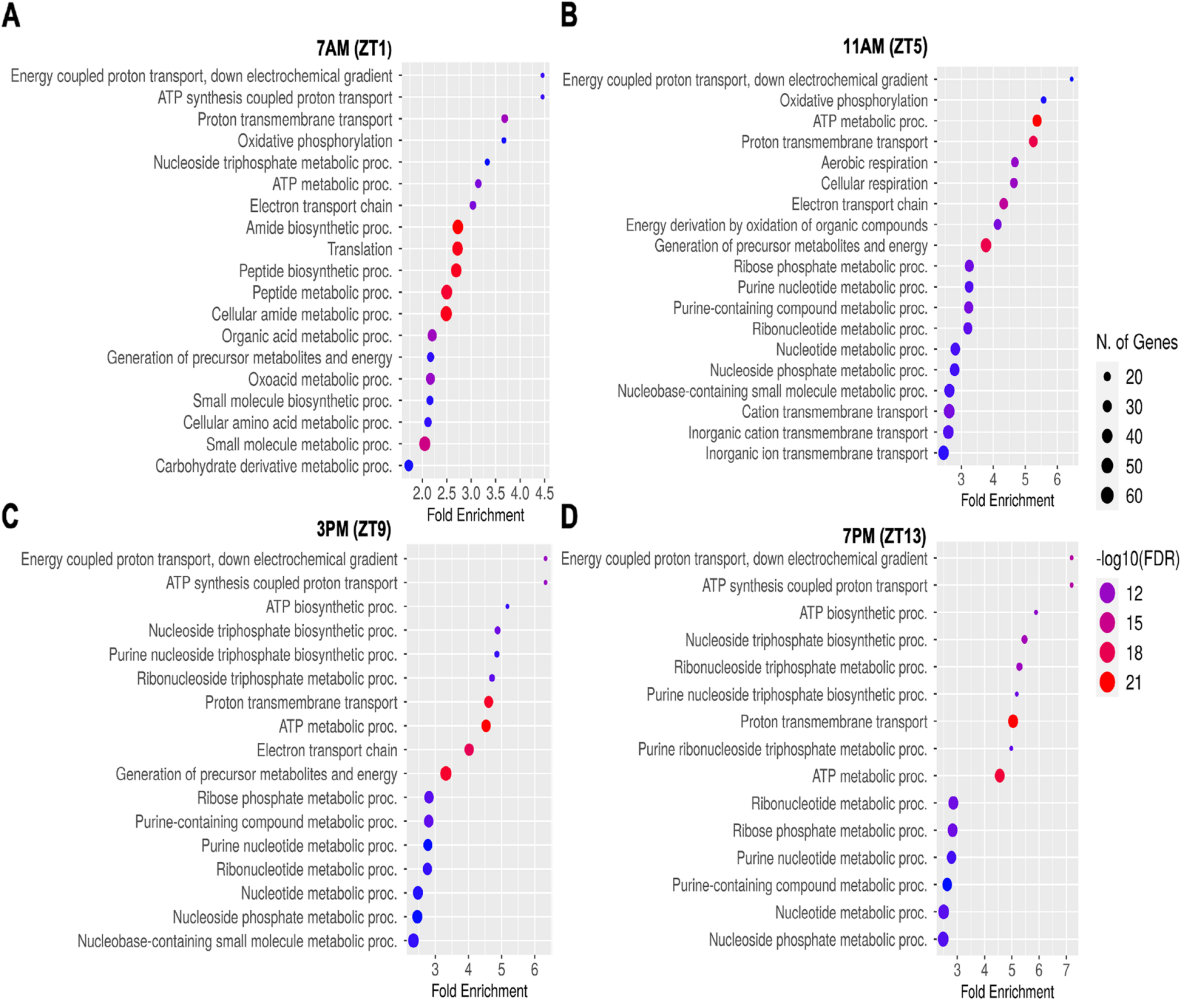
GO enrichment analysis of biological processes for up regulated differentially expressed genes at four different time points in a LD cycle (**A**) ZT1, (**B**) ZT5, (**C**) ZT9 and (**D**) ZT13. Only the top 20 were shown. The *x*-axis represents the proportion of genes that belong to a given functional category to the total number of differentially expressed genes. *p*-values were corrected using the Benjamini–Hochberg method

**Fig. 6:**
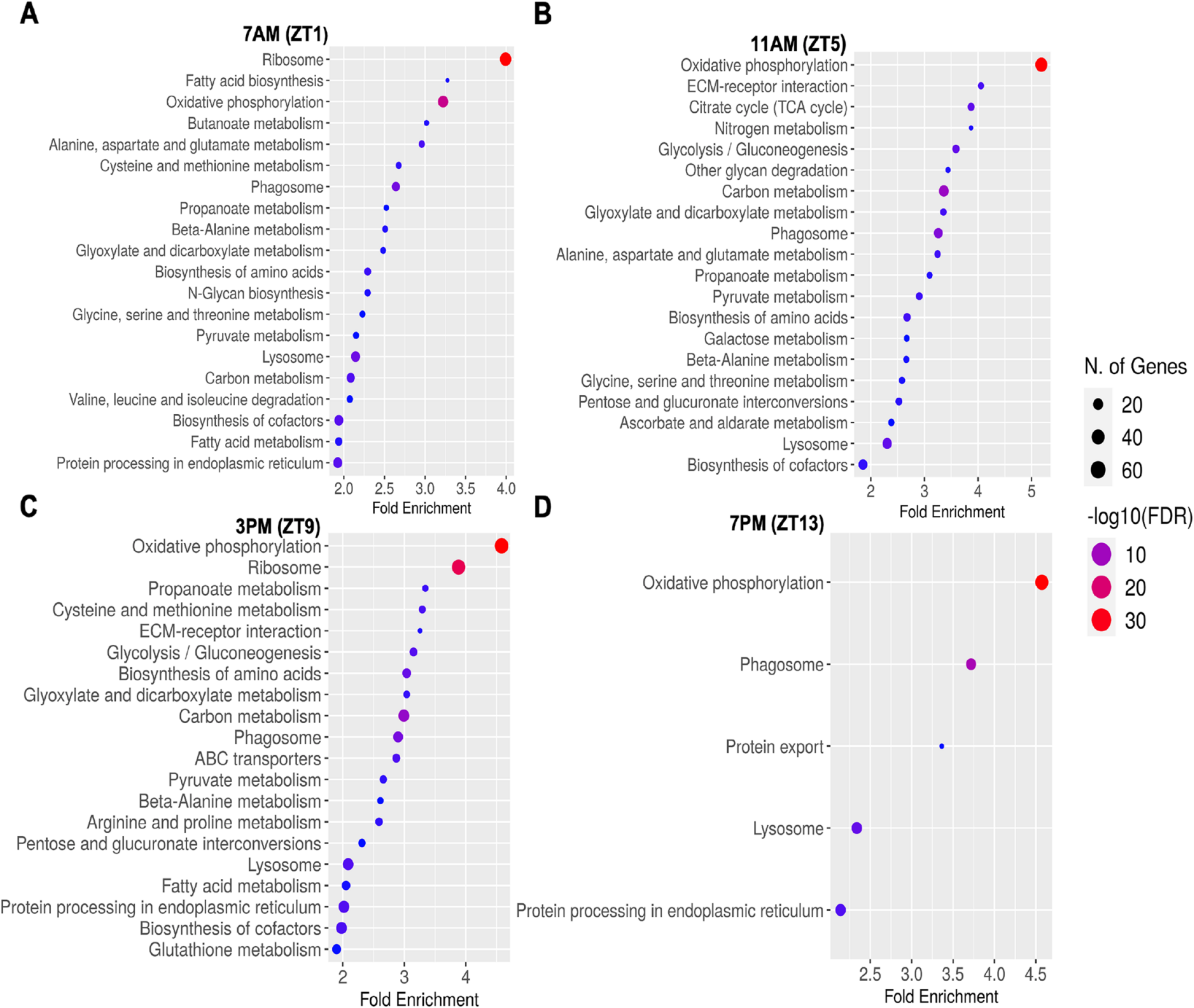
GO enrichment analysis of KEGG pathways for up regulated differentially expressed genes at four different time points in a LD cycle (**A**) ZT1, (**B**) ZT5, (**C**) ZT9 and (**D**) ZT13. *p*-values were corrected using the Benjamini–Hochberg method.

Additionally, separate GO enrichment analyses were performed for both upregulated and downregulated genes from the DEGs (P<0.05) at all four time points (Supplementary file 3: S6 to S9 and Supplementary file 4: S10 to S13). Both upregulated and downregulated genes with GO term sizes ranging from 10 to 500 were selected for GO enrichment analyses at all time points. The analysis of upregulated genes at four time points (ZT1, ZT5, ZT9, and ZT13) revealed significant enrichment in several biological processes (Fig. 5A to D). These processes included energy-coupled proton and proton transmembrane transports, ATP metabolic process, and various ribonucleotide and nucleotide metabolic processes, which were enriched at all time points. Furthermore, ZT1, ZT5, and ZT9 in a LD cycle showed enrichment in biological processes related to oxidative phosphorylation, electron transport chain, and nucleoside metabolic processes. KEGG analysis for upregulated DEGs identified common pathways enriched at all time points (Fig. 6A to D), including oxidative phosphorylation, phagosome, and protein export. Additionally, specific pathways were enriched at different time points, such as carbon metabolism and pyruvate metabolism at ZT1, ZT5, and ZT9; glycolysis/gluconeogenesis at ZT5 and ZT9; and fatty acid metabolism at ZT1 and ZT9.

**Fig. 7:**
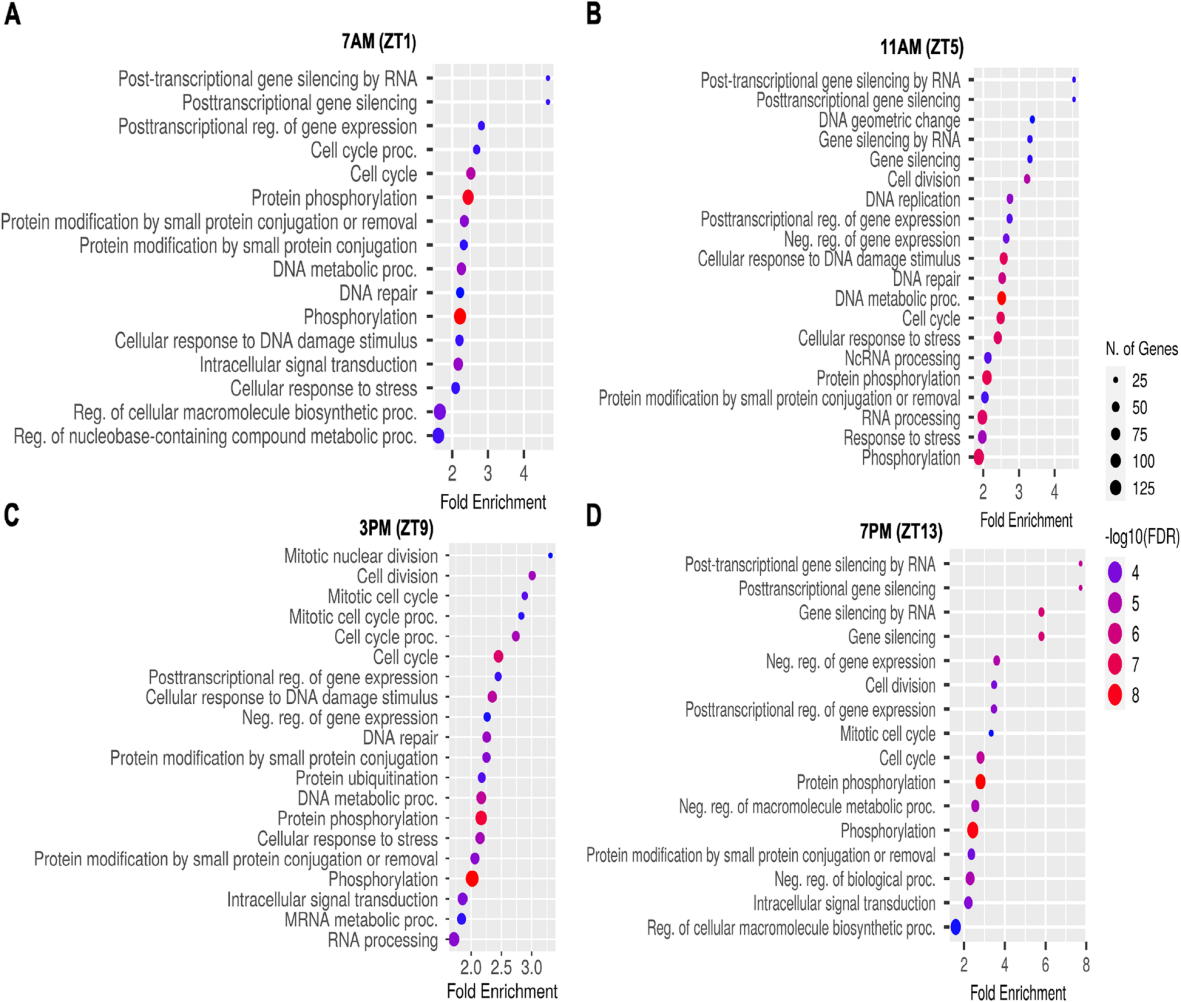
GO enrichment analysis of biological processes for down regulated differentially expressed genes at four different time points in a LD cycle (**A**) ZT1, (**B**) ZT5, (**C**) ZT9 and (**D**) ZT13. Only the top 20 were shown. The *x*-axis represents the proportion of genes that belong to a given functional category to the total number of differentially expressed genes. *p*-values were corrected using the Benjamini–Hochberg method

**Fig. 8:**
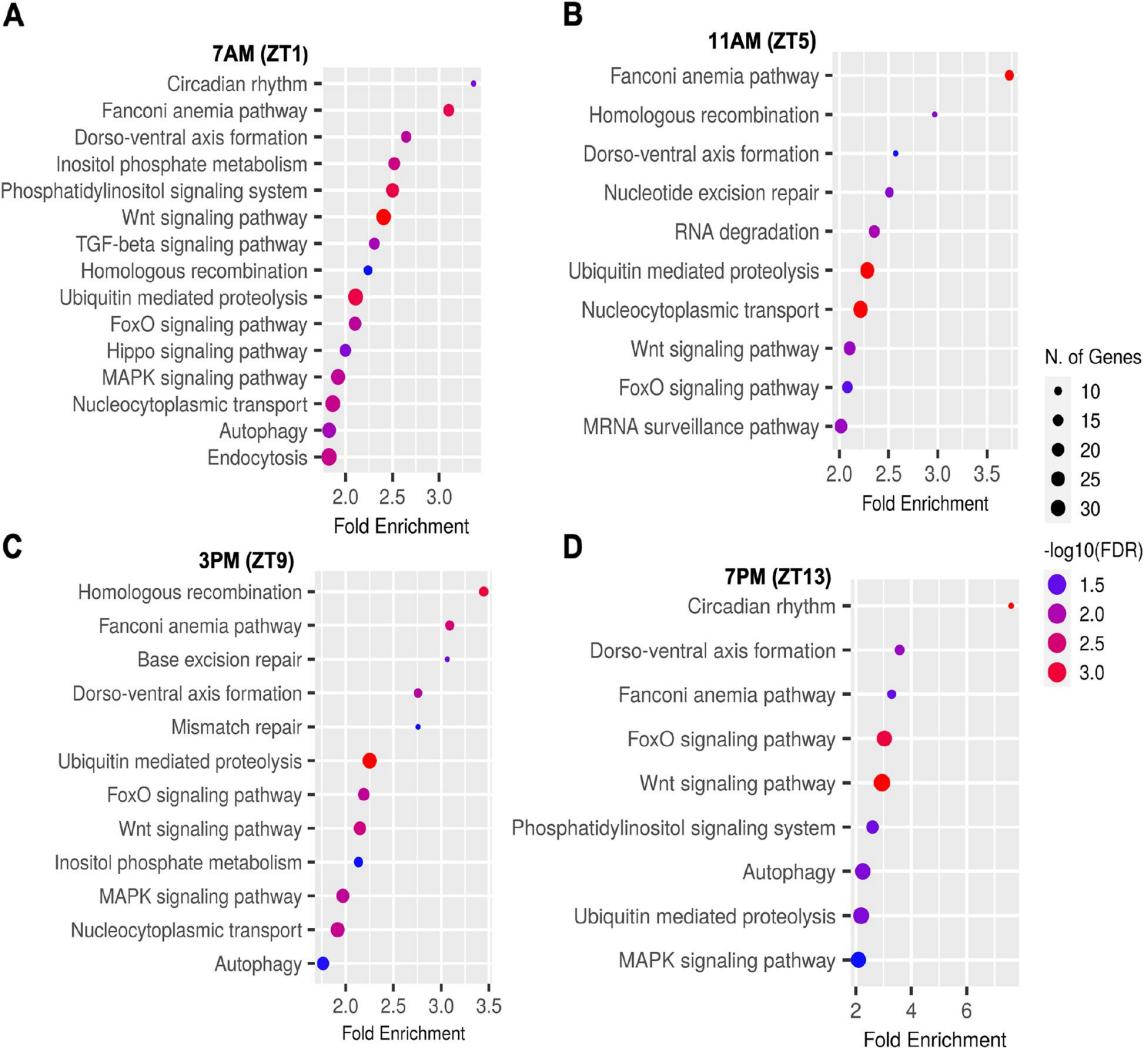
GO enrichment analysis of KEGG pathways for down regulated differentially expressed genes at four different time points in a LD cycle (**A**) ZT1, (**B**) ZT5, (**C**) ZT9 and (**D**) ZT13. *p*-values were corrected using the Benjamini–Hochberg method

For downregulated genes at the four time points, significant enrichment was observed in several molecular functions and biological processes (Fig. 7A to D). GO analysis revealed enrichment in post-transcriptional gene silencing by RNA, cell cycle, protein phosphorylation, cell division, negative regulation of biological process, DNA metabolic process, DNA repair, cellular response to stress, and cellular response to DNA damage stimulus at all time points. Additionally, downregulated genes were enriched in negative regulation of gene expression and regulation of cellular macromolecule biosynthetic process at ZT13. KEGG pathway analysis identified common pathways enriched in downregulated genes at all time points (Fig. 8A to D), including Fanconi anemia pathway, Dorso-ventral axis formation, FoxO signaling pathway, Wnt signaling pathway, and Ubiquitin-mediated proteolysis. Circadian rhythm and Phosphatidylinositol signaling pathway were impacted as a rhythmic pattern at both ZT1 and ZT13 time points only. Additionally, MAPK-related signaling pathway and Autophagy were enriched at ZT1, ZT9, and ZT13.

The GO enrichment analysis of biological processes for DEGs across different time points revealed significant downregulated genes related to peptidase activity and molecular function, cellular respiration, metabolic processes, glycolysis/gluconeogenesis, external stimulus, innate immune response, immune response, and response to other organisms and biotic stimuli. Notably, protein phosphorylation and developmental processes, circadian rhythm were prominently downregulated at the ZT13 time point when there was no light for the *Cyc* KO line. Additionally, cellular response to stress, metabolic pathways and DNA damage repair pathways were predominantly downregulated in the presence of light at ZT1, ZT5, and ZT9 in the *Cyc* KO line.

### Sensory perception and stimulus response

GO enrichment analyses of DEGs revealed arrhythmically expressed upregulated genes at all time points related to sensory perception (GO:0007600) and visual perception (GO:0007601), as well as nervous system processes (GO:0050877), which were enriched (Supplementary file 1: Table S4). Specifically, genes such as AAEL005621 (long wavelength-sensitive opsin), AAEL005625 (long wavelength-sensitive opsin), AAEL000596 (myosin), AAEL004006 (acetylcholine receptor protein alpha 1, 2, 3, 4 invertebrate), AAEL011174 (gustatory receptor Gr11), and AAEL000075 (gustatory receptor Gr9) were involved. Additionally, genes involved in the G-coupled receptor signaling pathway (GO:0007186) and detection of external stimuli (GO:0009581) were enriched, including AAEL000266 (GPCR Orphan/Putative Class A Family), AAEL000851 (G-protein coupled receptor), and AAEL007372 (GPCR GABA B Family). Furthermore, genes related to odorant binding proteins (GO:0005549), such as AAEL002587 (OBP11), AAEL002591 (OBP13), AAEL002617 (OBP12), AAEL005680 (Odorant receptor), and AAEL008013 (OBP38), etc., were differentially expressed at all time points arrhythmically in *AeCyc*^-/-^.

### Immune response

The GO enrichment analyses for DEGs arrhythmically expressed at ZT5, ZT9 and ZT13 showed that genes involved in immune pathways (Supplementary file 1: Table S4), such as AAEL007619 (Toll-like receptor), AAEL013441 (Toll-like receptor), AAEL009474 (Peptidoglycan Recognition Protein), AAEL003841 (Defensin-A), AAEL029047 (cecropin), and AAEL003389 (attacin), were upregulated rhythmically in *AeCyc*^-/-^.

### Enrichment map

**Fig. 9:**
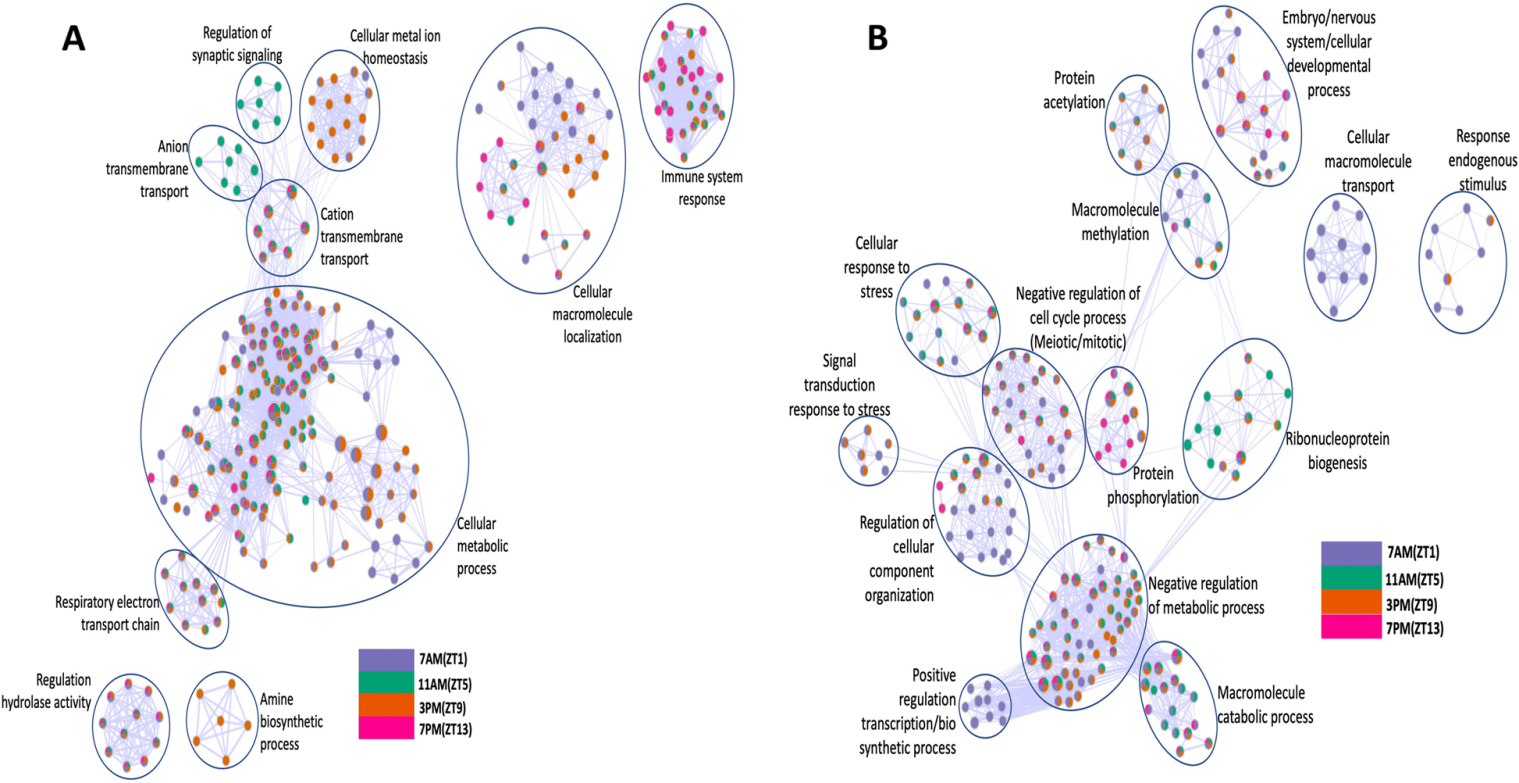
GO enrichment map of biological processes for differentially expressed **A**). up regulated genes **B**). down regulated genes. The enrichment network shows the pathway/gene ontology (GO) -biological processes (BP) gene sets (nodes) that are significantly associated (false discovery rate <0.05) with *AeCyc*^*-/-*^. The node color refers to the association with the different time points in a LD cycle; node size is proportional to the gene-set size. Edges connect related pathways/GO-BPs. Edge thickness is proportional to the similarity between 2 pathway/GO-BP

The biological processes affected by the up and downregulation of genes at different time points in a LD cycle were investigated, revealing a complex interplay between the circadian clock and other biological pathways in mosquitoes. The GO enrichment map analysis indicated that both up and downregulation of genes significantly impacted cellular and metabolic processes in mosquitoes (Fig. 9A and B). The BP enrichment map for upregulated genes highlighted a decrease in cellular metabolic processes, cellular macromolecule localization, immune system response, respiratory electron transport chain, synaptic signaling, ion transmembrane transport, etc. These clusters of functional groups correlated with differentially expressed genes mentioned in the heatmap and GO enrichment analysis. Interestingly, our results revealed that biological processes continued to function throughout the day. Specifically, at ZT1 (violet), ZT5 (green), and ZT9 (orange), there was extensive physiological activity observed during the day compared to ZT13 (magenta). It's noteworthy that the biological processes would remain operational in *AeCyc*^-/-^ under LD conditions without significant fitness defects.

For downregulated genes, the BP enrichment map showed enrichment related to various cellular and developmental processes (Fig. 9B). Specific clusters highlighted genes involved only at ZT1 and ZT9 time points, such as response to endogenous stimulus, signal transduction response to stress. Furthermore, gene sets from ZT1 largely showed in most biological clusters, such as cellular macromolecule transport, positive regulation of transcription, and regulation of cellular component organization. Negative regulation of metabolic and cell cycle processes was observed in all time points, predominantly during the daytime.

## Discussion

The present study investigated the impact of knockout of the core clock gene *cycle* on gene expression patterns and functional processes that provide valuable insights into the transcriptional regulation and rhythmic gene expression in *Ae. aegypti* mosquito's physiology and behavior. The study utilized transcriptome profiling and differential gene expression analysis to identify genes that are rhythmically expressed in response to the circadian clock and those specifically affected by the absence of the *Cyc* gene.

One of the key findings of this study is the identification of rhythmic genes that are independent of a functional clock, referred to as system-driven genes (Fig. 2A). These genes exhibit lower amplitudes and a bimodal phase distribution, suggesting that they are regulated by other biological mechanisms, possibly through the integration of different environmental cues. This echoes findings in other insects, like Drosophila and *Anopheles gambiae*, where a significant portion of rhythmic genes were found to be independent of the core circadian clock [2]. The identification of these system-driven genes in *Ae. aegypti* expands our comprehension of the intricate regulatory networks governing the mosquito's biology. The presence of system-driven rhythmic genes in *Ae. aegypti*, particularly enriched in pathways such as ribosome, oxidative phosphorylation, ubiquitin-mediated proteolysis, and Wnt signaling, unveils a nuanced temporal regulation of cellular processes. Notably, in the absence of the canonical circadian cycle gene *AeCyc*^-/-^, certain circadian core clock genes persist in rhythmicity, suggesting a potential influence of system-driven genes on circadian maintenance. These findings resonate with insights from diverse organisms, exemplified by wild-type monarch butterflies, highlighting temporal coordination in key enzyme-encoding genes related to glucose, trehalose, and glycogen metabolism [12]. Similar patterns in Antarctic krill [13] and Drosophila heads [14] suggest a homeostatic mechanism ensuring rhythmic fuel production. In monarchs, a proposed mechanism involving pyruvate conversion in brain glial cells suggests a strategy for neuronal fuel supply during the active phase, emphasizing the intricate nature of circadian regulation in glucose metabolism [12]. Furthermore, in mice liver, circadian regulation in glucose-related genes, transcription, glycogen metabolism, phosphorylation, and Wnt signaling underscores the broader impact of circadian rhythms on metabolic processes. The observed differential expression of glucose-related genes in the muscle clock further highlights circadian regulation's centrality in governing glucose metabolism [15]. The identification of enriched pathways in *Ae. aegypti*, coupled with the context of *AeCyc*^-/-^ and the persistence of rhythmicity in specific core clock genes, underscores the conserved and intricate nature of circadian regulation in metabolic processes. These findings lay a robust foundation for understanding the molecular mechanisms governing rhythmic gene expression and their physiological implications across diverse organisms, advancing our comprehension of the intricate interplay between system-driven and circadian-driven rhythmicity.

Another important finding is the impact of the *Cyc* knockout on the expression of circadian clock genes. The loss of *Cyc* resulted in the arrhythmic expression of essential clock genes, such as *Clk*, *Cry1*, and *Cry2*. However, *Per* and *Vri* maintained rhythmic expression, albeit with lower amplitudes and significantly reduced levels at ZT13. Additionally, other clock genes such as *Tim*, *Pdp1*, and *Cwo* maintained rhythmic expression without any significant differences. This profile aligns with our earlier study on *AeCyc*^-/-^ induced endogenous clock genes expression, also substantiating the connection between disrupted clock genes and shifts in *Ae. aegypti* mosquito's fitness, feeding behavior, mating, and daily rhythms [16]. The differential gene expression analysis between *Cyc*^*-/-*^ and wild-type mosquitoes revealed thousands of genes that are differentially expressed at distinct time points in a light-dark cycle. These findings indicate that the absence of *Cyc* not only affects the core clock machinery but also has widespread effects on the mosquito's transcriptome. Many of the differentially expressed genes were associated with metabolic processes, signaling pathways, and immune responses emphasizing the indispensable role of the circadian clock in orchestrating the mosquito's physiological landscape. It's worth mentioning that the earlier study demonstrated that AeCYC^−/−^ was unable to form a functional heterodimer with CLK [11]. In Drosophila melanogaster, CYC/CLK heterodimer plays an essential role in the core circadian feedback loop that triggers the transcription of *per* and *tim* [17]. This concurs with recent findings that disrupted circadian neuronal circuits in *Ae. aegypti* exhibit substantially reduced PER protein levels under constant light conditions [3]. These findings highlight that *tim* and *per* expression peaks during the early night, instigated by the feedback loop of the CYC/CLK heterodimer [17]. Our present transcriptomic analysis of *AeCyc*^*-/-*^ validate the earlier endogenous circadian clock genes expression findings in *AeCyc*^*-/-*^ line [11], a significant reduction in *per* expression at ZT 13 becomes evident in *AeCyc*^-/-^ compared to WT during the early night. This confirms that despite the absence of the CYC/CLK heterodimer activator in *AeCyc*^-/-^ mosquitoes, which led to reduced expression of *per* and *tim* genes, their expression persisted, suggesting the existence of alternative mechanisms in the absence of the CYC/CLK heterodimer activator.

The GO enrichment analysis further supported the importance of the circadian clock in regulating various biological processes in *Ae. aegypti*. The enrichment of genes involved in DNA topological change, metabolic processes, and protein phosphorylation in *AeCyc*^-/-^ specific rhythmic genes underscores the clock's pivotal role in coordinating cellular activities and environmental responses. The KEGG pathway enrichment analysis further revealed that the Fanconi anemia pathway, MAPK signaling pathway, and ubiquitin-mediated proteolysis were among the most enriched rhythmic pathways in the *AeCyc*^-/-^ specific *Ae. aegypti*. This parallels findings in *D. melanogaster* where the p38 MAP Kinase of the MAPK pathway is a core element of the circadian clock and a player in stress-input pathways [16]. Similarly, in mammalian cells, MAPKs exhibit diverse roles in circadian clock functioning, acting as circadian oscillators via BMAL1/CLK complex phosphorylation [18]. The current study spotlights that the MAPK pathway and circadian rhythm pathways in *AeCyc*^-/-^ are downregulated throughout the LD cycle, particularly at ZT1, ZT9, and ZT13. On the flip side, the enrichment of fatty acid biosynthesis, longevity-regulating pathways, and metabolic pathways in WT-specific rhythmic genes indicates the clock's involvement in lipid metabolism and longevity regulation. The outcomes of this study also illuminate the mosquito's sensory perception and stimulus response. Differential gene expression analysis reveals upregulated genes involved in visual perception, the G-coupled receptor signaling pathway, odorant binding proteins, and the detection of external stimuli. These findings suggest that the circadian clock might influence the mosquito's ability to detect and respond to environmental cues such as light and odorants. Notably, several OBPs upregulated such as OBP11, OBP12, OBP13, OBP22, and OBP38, exhibited upregulation at ZT1 and ZT9, with subsequent multiple OBPs upregulated at ZT5 in light phase. Interestingly, only two odorant binding proteins were upregulated at ZT13 (Supplementary file 3). In line with previous findings, *AeCyc*^-/-^ mosquitoes demonstrate sustained flight activity throughout the light phase, deviating from the typical bimodal pattern seen in wildtype mosquitoes. The *AeCyc*^-/-^ mosquitoes exhibited an overall increase in activity, with a minor peak at ZT0 and a prominent peak towards the end of the light phase. Additionally, during the dark phase, *AeCyc*^-/-^mosquitoes predominantly became inactive, mirroring the behavior of wildtype mosquitoes [11]. These outcomes suggest a disruption in the controlled expression of OBPs, which typically exhibits a bimodal pattern regulated by the circadian clock in *AeCyc*^-/-^mosquitoes. Previous research emphasized the intricate interplay between clock-derived and photic signals in regulating OBPs [2]. In insects, antennal clocks are believed to modulate olfactory reception within the antennae themselves [19, 20], with limited evidence suggesting a direct influence on brain mechanisms [21]. Particularly, OBP22, which demonstrated consistent upregulation throughout the LD cycle, was identified as crucial for flight activity and blood-feeding in *Ae. aegypti* [22]. Intriguingly, our prior study revealed that *AeCyc*^-/-^mosquitoes exhibited a reduced response to human host odor. However, the current whole-body transcriptomic profiling failed to detect any specific downregulation of OBPs correlated with host odor response. Therefore, an antennal-specific transcriptomic analysis of the *AeCyc*^-/-^ line is warranted to gain further insights into the role of clock-modulated chemosensory gene expression. However, interestingly, the identified differential upregulation in pathways related to proton transport, oxidative phosphorylation, and the electron transport chain aligns with previous findings in insects, particularly mosquitoes. Insects, including mosquitoes, rely heavily on precise regulation of energy metabolism to support their diverse activities, ranging from flight to reproductive processes. Studies in mosquitoes have demonstrated the significance of these pathways in orchestrating energetic balance. For instance, research on *Ae. aegypti* mosquitoes has highlighted the importance of oxidative phosphorylation in meeting the energy demands associated with flight and other essential physiological functions [23]. Additionally, investigations into the circadian regulation of metabolic pathways in *Anopheles gambiae*, another important mosquito species, have indicated the involvement of proton transport and electron transport chain components [24]. The potential impacts of the observed differential gene expression on locomotor activity profiles in the context of wild-type (WT) and *AeCyc*^-/-^mosquitoes resonate with broader studies on circadian rhythms and behavior in insects. Previous work in Drosophila has revealed intricate connections between the circadian clock, energy metabolism, and locomotor activity [25]. These insights provide a valuable framework for understanding how disruptions in the circadian clock, as observed in *AeCyc*^-/-^ mosquitoes, might influence energy-related pathways and subsequently impact behavioral patterns. Hence, this differential expression of genes involved in enrichment of pathways such as proton transport, oxidative phosphorylation, and the electron transport chain in energy metabolism offers a unique perspective on the potential repercussions for locomotor activity profiles, drawing parallels with established knowledge in insect physiology and circadian regulation.

Additionally, our present study reveals differential gene expression in various visual and sensory perception genes, including long-wavelength opsins. Similar rhythmic expression of opsins has been observed in several insects, including mosquitoes, where opsins play a role in regulating the circadian clock in a light-dark cycle [2, 26, 27]. The entrainment pathway for environmental cues (photoreceptor), the central oscillator (central clock), and the output pathway translating information into behavioral and physiological rhythms [28] all contribute to the regulation of retinal physiology by the circadian clock, as indicated by the extensive targeting of genes involved in phototransduction and development [29]. The upregulated expression of clock genes without rhythm in *AeCyc*^*-/-*^ mosquitoes in this study might also influence the expression of long-wavelength-sensitive opsins. Notably, in zebrafish, functional *Clock* gene expression, a key component of the central circadian pacemaker, maintains the circadian rhythm of LC opsin mRNA expression through cyclic adenosine monophosphate (cAMP)-dependent signaling pathways [30]. Given the substantial variations in ambient light between day and night, organisms must adjust their visual systems' sensitivity to cope [31]. In the context of mosquitoes, multiple genes in the visual transduction pathway display rhythmic expression [2, 24], corresponding to elevated nocturnal rhodopsin levels in the eye [32]. Similarly, Anopheles mosquitoes, which are nocturnal host-seekers, elevate their olfactory system activity at night. It was demonstrated that gene expression and protein levels of odorant binding proteins (OBPs) peak around dusk, while heightened olfactory sensitivity to hydrophobic compounds during the night [2, 24]. The regulation of olfactory responses by G protein-coupled receptor kinases (GPRKs) and odorant receptors (ORs) is well-established in Drosophila, where their expression and functions are influenced by the circadian clock [33]. Olfaction is crucial for mosquitoes, enabling the detection of blood-feeding hosts, sources of sugar feeding, and suitable oviposition sites [34, 35].

Furthermore, the enrichment of immune-related genes in the DEGs at specific time points underscores the circadian clock's potential influence on the mosquito's immune response. This aligns with studies in other insects, linking circadian rhythms with immune functions [36]. The GO enrichment analyses for differentially expressed genes (DEGs) at ZT5, ZT9, and ZT13 provide intriguing insights into the temporal regulation of immune pathways in *AeCyc*^*-/-*^ mosquitoes. The arrhythmic expression of immune-related genes like Toll-like receptors, Peptidoglycan Recognition Protein, Defensin-A, cecropin, and attacin in *AeCyc*^*-/-*^ mosquitoes, compared to wildtype *Ae. aegypti*, extends our comprehension of the impact of disrupted circadian rhythms on vector competence. This aligns with the established role of the circadian clock in modulating diverse physiological processes, including immune responses [36]. Synthesizing these results with earlier studies enriches our grasp of how disrupted circadian rhythms influence vector competence. Prior studies underscored the rhythmic expression of immune genes in mosquitoes and their potential impact on malaria infection modulation [24, 37, 38]. This rhythm intricately intertwines with the circadian clock, which synchronizes physiological activities with the external environment [39] The circadian clock ensures that activities like feeding, metabolism, and immunity harmonize with the day-night cycle. The rhythmic expression of immune genes, including Imd and melanization pathway participants, optimizes the mosquito's ability to ward off pathogens at specific times of the day with higher exposure potential [2]. However, our study's findings, displaying disrupted rhythms in immune gene expression in *AeCyc*^*-/-*^ mosquitoes, signify that an absent functional circadian clock could upset immune response regulation, carrying weighty implications for vector competence. The discovery of immune gene arrhythmic expression in *cycle* knockout *AeCyc*^*-/-*^ mosquitoes establishes a crucial bridge between perturbed circadian rhythms and altered vector competence. Integrating these findings with the earlier study's insights on immune rhythms, ROS detoxification, and gut microbiota, a more comprehensive picture emerges of how circadian clock disruption impacts the mosquito's ability to transmit vector borne diseases [40, 41].

## Conclusions

In summary, this study comprehensively examines the *Cyc*^-/-^ *Ae. aegypti* transcriptome, revealing responses to circadian clock influence, including the discovery of system-driven genes operating independently of the core clock machinery. The disruption of the *Cyc* gene triggers a cascade of gene expression changes, affecting essential clock genes, metabolic processes, signaling pathways, and immune responses, with a notable GO enrichment in odorant binding proteins, accentuating the clock's impact on sensory perception. Moreover, disrupted circadian clocks link to altered mosquito host-seeking behavior, evidenced by altered *AeCyc*^*-/-*^ locomotor activity pattern and reduced response to human host odor during peak hours [11], implying significant consequences for vectorial capacity [42, 43]. This emphasizes the significance of the circadian clock and its role in shaping the mosquito's ability for disease transmission. These insights into the molecular basis of daily rhythms provide valuable perspectives for mosquito biology and control research.

## Materials and methods

### Mosquito rearing

*Ae. aegypti* (Liverpool strain) wildtype and *cycle* knockout lines were reared under controlled laboratory conditions at a temperature of 27 °C and a relative humidity of 60-70%. The mosquitoes followed a 12:12 h light/dark cycle, which included 1-hour transitions for dawn and dusk. Eggs were hatched in deionized water, and the resulting larvae were provided with daily feedings of ground Tetramin® fish food. Adult mosquitoes were offered cotton balls soaked in a 10% sucrose solution. Adult females were fed on defibrinated sheep blood provided through an artificial membrane feeder.

### Sample preparation and RNA extraction

A total of six replicates (*n*= 10 mosquitoes per replicate) for both the wildtype control group and the *AeCyc*^-/-^ group mosquitoes were collected five days after emergence at four different time points in a day/light cycle i.e. ZT1, ZT5, ZT9 and ZT13.

Female mosquitoes were immobilized by placing them at −20°C for about a minute, transferred to petri-plates on ice and immediately stored in RNAlater***®*** Ice (Ambion). Samples were stored at −80°C and RNA extraction was carried out for all samples within 48 h. Total RNA was isolated from each sample using miRNeasy (Qiagen) columns using the protocol provided by Qiagen. A Qubit fluorometer (Life Technologies) was used to quantify RNA initially, as well as a NanoDrop spectrophotometer (Thermo Scientific) followed by a quality assessment with an RNA Pico LabChip analysis on an Agilent BioAnalyzer 2100 (Agilent Technologies) by the Agrilife Genomics Center at Texas A&M University. Messenger RNA was enriched from about 1 μg of total RNA using the Poly-A enrichment method and cDNA libraries were prepared using an Illumina TruSeq RNA Library kit (Illumina). Preparation and sequencing of libraries were both performed at the AgriLife Genomics core facility at Texas A&M University, College Station, Texas. All libraries were sequenced over two lanes of Illumina Novaseq 6000 platform running in high output mode and generating 50 bp paired end reads. A sequencing depth of approximately 80 million paired end reads per sample was generated for each replicate and used for further analysis.

### RNA sequence analysis

The resulting sequences were evaluated for quality using FastQC (version 0.11.9.). Paired end adaptors were trimmed using TrimGalore v.0.6.6. The *Ae. aegypti* genome sequence (version: AaegL5_2) from Vectorbase was used for mapping and alignments by HISAT2 (version 2.2.1). Raw read indexing and sorting alignments were performed using SAMtools (version 1.11) and count files were generated using HTseq (version 0.11.3). High performance computing resources at Texas A&M University (http://hprc.tamu.edu) were used for quality control analyses, reference-based read alignment and generating count files.

### Statistical and differential gene expression analyses

Tests for differential expression were performed in the R package DESeq2 in order to identify upregulated/downregulated genes. The Benjamini-Hochberg approach was used to adjust *P*-values for multiple testing. For the selection of differentially expressed (DE) genes, we used a false discovery rate (FDR) adjusted *p*-value (i.e., q-value) of < 0.05. For the analysis of rhythmicity from time-series gene expression analysis for two different conditions (*AeCyc*^-/-^ and WT), we used *dryR* (for Differential Rhythmicity Analysis in R: https://github.com/naef-lab/dryR) to assess differential rhythmicity [15]. Count files were used to estimate changes in the parameter amplitude (log_2_ fold-change peak-to-trough), phase (time of peak), and mean expression levels for datasets via *dryR*.

### Gene ontology analysis and enrichment map

Gene ontology analysis was performed for rhythmic genes from *AeCyc*^-/-^ and WT specific, also both up and down regulated DEGs using ShinyGO 0.77/ gProfiler using significance threshold to Benjamini-HochBerg FDR to < 0.05. Gene Ontology category, BP:biological process, MF: molecular function and CC: cellular component, KEGG pathway were generated. A gene set size or pathway size minimum 10 and maximum 500 filtered to exclude gene-sets more than 500 genes. A graphical display of gene-sets that passed the significance threshold of 0.05 for the gene-set databases that we have selected, GO Biological process (GO:BP) and KEGG Pathway. Both up and down regulated genes were used to produce enrichment maps using Cytoscape (v3.9.1). GO enrichment gmt files were generated from gProfiler (https://biit.cs.ut.ee/gprofiler/gost) used to visualize the enrichment map using Cytoscape. Gene-sets were grouped according to their mutual overlap, and clusters were manually circled and labeled to highlight the impact of *AeCyc*^-/-^ on the prevalent biological functions among related gene-sets at different time points in a LD cycle.

### Supplementary Information

**Additional file 1:** **Table S1.** Summary statistics of sequencing data for AeCyc-/-and WT transcriptome analysis including mapping totals, Q20 and GC percentage. **Table S2.** Differentially expressed up and down regulated genesshowing from volcano plot at four different time points in a LD cycle. **Table S3.** Differentially expressed genes in a heatmap (>5-fold change) at four different time points in a LD cycle. **Table S4.** Differentially expressed genes GO enriched at four different time points in a LD cycle, specifically for GO:BP, Sensory perception (GO:0007600), Nervous System Process (GO:0050877), G protein-coupled receptor signaling pathway (GO:0007186), Odorant binding (GO:0005549), Immune response (GO:0006955), etc. **Figure S1.** Gene ontology (GO) enrichment analysis for rhythmically differentially expressed genes in *Cyc* KO-specific and WT-specific groups. (A) Enriched GO term biological processes for *Cyc* KO-specific genes. (B) Enriched KEGG pathways for *Cyc* KO-specific genes. (C) Enriched GO term biological processes for WT specific genes. (D) Enriched KEGG pathways for WT specific genes. Only the top 20 were shown. The *x*-axis represents the proportion of genes that belong to a given functional category to the total number of differentially expressed genes. *p*-values were corrected using the Benjamini–Hochberg method.

**Additional file 2:** **Supplementary file S5.** Model results of differential rhythmicity in time-series gene expression for two conditions Cyc KO and WT. **Supplementary file S5.1.** Cyc KO specific genes showed differential rhythmicity. **Supplementary file S5.2.** WT specific genes showed differential rhythmicity. **Supplementary file S5.3.** GO enrichment anlysis for Cyc KO specific genes. **Supplementary file S5.4.** GO enrichment anlysis for WT specific genes.

**Additional file 3:** **Supplementary file S6.** GO enrichment analysis for up regulated genes at ZT1 time point. **Supplementary file S7.** GO enrichment analysis for up regulated  genes at ZT5 time point. **Supplementary file S8.** GO enrichment analysis for up regulated  genes at ZT9 time point. **Supplementary file S9.** GO enrichment analysis for up regulated  genes at ZT13 time point.

**Additional file 4:** **Supplementary file S10.** GO enrichment analysis for down regulated  genes at ZT1 time point. **Supplementary file S11.** GO enrichment analysis for down regulated  genes at ZT5 time point. **Supplementary file S12.** GO enrichment analysis for down regulated  genes at ZT9 time point. **Supplementary file S13.** GO enrichment analysis for down regulated  genes at ZT13 time point.

## Data Availability

The datasets generated and analyzed during the current study are available in the Gene Expression Omnibus (GEO) repository GEO accession: GSE241953**.**

## Abbreviations

*AeCyc*^-/-^: *Aedes aegypti cycle* homozygous knock out line
bp: Base pair
BP: Biological process
cAMP: Cyclic adenosine monophosphate
CC: Cellular component
Clk: Clock
Cry1: Cryptochrome-1
Cry2: Cryptochrome-2
Cwo: Clockworkorange
Cyc: Cycle
DE: Differential expression
DEGs: Differentially expressed genes
FDR: False discovery rate
GO: Gene Ontology
GPCR: G-Protein coupled receptors
GPRKs: G protein-coupled receptor kinases
Imd: Immune deficiency
KEGG: Kyoto Encyclopedia of Genes and Genomes
KO: Knock out
LD: Light and dark
MAPK: Mitogen-activated protein kinase
MF: Molecular function
OBPs: Odorant binding proteins
ORs: Odorant receptors
Pdp1: Par domain protein 1
Per: Period
ROS: Reactive oxygen species
Tim: Timeless
Vri: Vrille
WT: Wildtype
ZT: Zeitgeber
