# Supplementary material for: Effects of circadian clock disruption on gene expression and biological processes in Aedes aegypti
Source: BMC Genomics. 2024 Feb 13;25:170. doi: 10.1186/s12864-024-10078-8 (PMC10863115; doi:10.1186/s12864-024-10078-8)
Supplement: Supplementary file 1 — Additional file 1: Table S1. Summary statistics of sequencing data for AeCyc-/-and WT transcriptome analysis including mapping totals, Q20 and GC percentage. Table S2. Differentially expressed up and down regulated genesshowing from volcano plot at four different time points in a LD cycle. Table S3. Differentially expressed genes in a heatmap (>5-fold change) at four different time points in a LD cycle. Table S4. Differentially expressed genes GO enriched at four different time points in a LD cycle, specifically for GO:BP, Sensory perception (GO:0007600), Nervous System Process (GO:0050877), G protein-coupled receptor signaling pathway (GO:0007186), Odorant binding (GO:0005549), Immune response (GO:0006955), etc. Figure S1. Gene ontology (GO) enrichment analysis for rhythmically differentially expressed genes in Cyc KO-specific and WT-specific groups. (A) Enriched GO term biological processes for Cyc KO-specific genes. (B) Enriched KEGG pathways for Cyc KO-specific genes. (C) Enriched GO term biological processes for WT specific genes. (D) Enriched KEGG pathways for WT specific genes. Only the top 20 were shown. The x-axis represents the proportion of genes that belong to a given functional category to the total number of differentially expressed genes. p-values were corrected using the Benjamini–Hochberg method. [file 12864_2024_10078_MOESM1_ESM.docx]

| **Samples** | **Read length (Filtered %)** | **Total read mapped (%)** | **Q20 (%)** | **GC (%)** |
| --- | --- | --- | --- | --- |
| *AeCyc^-/-^* ZT1(7AM) | 99.5 | 98.53 | 99.47 | 47 |
| *AeCyc^-/-^* ZT5(11AM) | 99.4 | 99.10 | 99.57 | 47 |
| *AeCyc^-/-^* ZT9(3PM) | 99.5 | 99.84 | 99.47 | 47 |
| *AeCyc^-/-^* ZT13(7PM) | 99.5 | 99.54 | 99.48 | 48 |
| WT ZT1(7AM) | 99.5 | 98.84 | 99.47 | 48 |
| WT ZT5(11AM) | 99.5 | 99.90 | 99.47 | 48 |
| WT ZT9(3PM) | 99.4 | 99.52 | 99.47 | 48 |
| WT ZT13(7PM) | 99.4 | 99.43 | 99.53 | 48 |

Table S1: Summary statistics of sequencing data for *AeCyc^-/-^*and WT transcriptome analysis including mapping totals, Q20 and GC percentage.

Table S2: Differentially expressed up and down regulated genes showing from volcano plot at four different time points in a LD cycle.

| **7AM (ZT1)** | **DOWNREGULATED GENES** | | |
| --- | --- | --- | --- |
|  | **Gene IDs** | **Product description** | **Pfam description** |
|  | AAEL023559 | unspecified product | Peptidase M2, peptidyl-dipeptidase A |
|  | AAEL025123 | unspecified product | GRIP domain |
|  | AAEL001442 | map-kinase activating death domain protein (madd)/denn/aex-3(*c. elegans*) | cDENN domain;uDENN domain |
|  | AAEL008678 | unspecified product | Calponin homology domain; EB1, C-terminal |
|  | AAEL002753 | zinc finger protein | Zinc finger C2H2-type |
|  | AAEL010461 | unspecified product | Peroxisome membrane protein, Pex16 |
|  | AAEL000484 | unspecified product | Protein of unknown function DUF745 |
|  | | | |
| **11AM (ZT5)** | AAEL023559 | unspecified product | Peptidase M2, peptidyl-dipeptidase A |
|  | AAEL002049 | *Cycle* | Myc-type, basic helix-loop-helix (bHLH) domain |
|  | AAEL008678 | unspecified product | Calponin homology domain; EB1, C-terminal |
|  | AAEL012832 | cytochrome B561 | Cytochrome b561/ferric reductase transmembrane |
|  | AAEL025123 | unspecified product | GRIP domain |
|  | AAEL006953 | unspecified product | Chitin binding domain |
|  | AAEL005893 | CRC domain-containing protein | CRC domain |
|  | AEL0001776 | unspecified product | N/A |
|  | AAEL011173 | unspecified product | Rab-GTPase-TBC domain |
|  | AAEL020535 | unspecified product | N/A |
|  | AAEL000058 | unspecified product | N/A |
|  | AAEL010461 | unspecified product | Peroxisome membrane protein, Pex16 |
|  | AAEL027886 | unspecified product | N/A |
|  | AAEL027327 | unspecified product | DDE superfamily endonuclease domain;HTH CenpB-type DNA-binding domain;DNA binding HTH domain, |
|  | AAEL022585 | unspecified product | N/A |
|  | AAEL022563 | unspecified product | N/A |
|  | | | |
| **3 PM (ZT9)** | AAEL023559 | unspecified product | Peptidase M2, peptidyl-dipeptidase A |
|  | AAEL025123 | unspecified product | GRIP domain |
|  | AAEL009123 | cytochrome P450 | Cytochrome P450 |
|  | AAEL002693 | venom allergen | CAP domain |
|  | AAEL012832 | Cytochrome B561 | Cytochrome b561/ferric reductase transmembrane |
|  | AAEL010461 | unspecified product | Peroxisome membrane protein, Pex16 |
|  | AAEL004585 | unspecified product | N/A |
|  | AAEL025347 | unspecified product | N/A |
|  | AAEL000397 | unspecified product | N/A |
|  | | | |
| **7PM (ZT13)** | AAEL004585 | unspecified product | N/A |
|  | AAEL025654 | unspecified product | N/A |
|  | AAEL020099 | unspecified product | N/A |
|  | | | |
| **7AM (ZT1)** | **UPREGULATED GENES** | | |
|  | **Gene IDs** | **Product description** | **Pfam description** |
|  | AAEL002800 | DNA polymerase epsilon | - |
|  | AAEL002404 | receptor protein tyrosine kinase | - |
|  | AAEL021832 | unspecified product | N/A |
|  | AAEL028994 | unspecified product | N/A |
|  | | | |
| **11AM (ZT5)** | AAEL002800 | DNA polymerase epsilon, catalytic subunit | - |
|  | AAEL007601 | trypsin | - |
|  | AAEL0204664 | unspecified product | N/A |
|  | | | |
| **3PM (ZT9)** | AAEL002800 | DNA polymerase epsilon, catalytic subunit | - |
|  | AAEL006859 | Myb-interacting protein, putative | - |
|  | AAEL017985 | unspecified product | N/A |
|  | AAEL001693 | serine-type enodpeptidase | - |
|  | | | |
| **7PM (ZT13)** | AAEL003211 | Beta-carotene dioxygenase | - |
|  | AAEL006451 | Unspecified product | - |
|  | AAEL005437 | transient receptor potential channel | - |
|  | AAEL005641 | C-Type Lectin (CTL) - galactose binding. | - |
|  | AAEL014516 | metalloproteinase, putative | - |
|  | AAEL004239 | unspecified product | N/A |
|  | AAEL001674 | serine-type enodpeptidase | - |
|  | AAEL009356 | unspecified product | Protein of unknown function DUF2373 |

Table S3: Differentially expressed genes in a heatmap (>5-fold change) at four different time points in a LD cycle.

| **Differentially expressed genes at four different time points in LD cycle** | | |
| --- | --- | --- |
| **Gene Id** | **Product Description** | **Computed GO Processes** |
| **Sensory perception (GO:0007600)** | | |
| AAEL000075 | gustatory receptor Gr9 | taste receptor activity |
| AAEL005621 | long wavelength sensitive opsin | G protein-coupled receptor activity;photoreceptor activity |
| AAEL005625 | long wavelength sensitive opsin | G protein-coupled receptor activity;photoreceptor activity |
| AAEL005680 | Odorant receptor [Source:UniProtKB/TrEMBL;Acc:Q179B4] | odorant binding;olfactory receptor activity |
| AAEL006259 | long wavelength sensitive opsin | G protein-coupled receptor activity;photoreceptor activity |
| AAEL006685 | G-protein, gamma-subunit, putative | GTPase activity |
| AAEL009991 | myosin iii | N/A |
| AAEL010416 | unspecified product | ATP binding; actin binding;cytoskeletal motor activity;nucleotide binding;protein binding;protein kinase activity |
| AAEL011174 | gustatory receptor Gr11 | taste receptor activity |
| AAEL005621 | long wavelength sensitive opsin | G protein-coupled receptor signaling pathway;phototransduction;protein-chromophore linkage;response to stimulus;signal transduction;visual perception |
| AAEL005625 | long wavelength sensitive opsin | G protein-coupled receptor signaling pathway;phototransduction;protein-chromophore linkage;response to stimulus;signal transduction;visual perception |
| AAEL006259 | long wavelength sensitive opsin | G protein-coupled receptor signaling pathway;phototransduction;protein-chromophore linkage;response to stimulus;signal transduction;visual perception |
| AAEL009991 | myosin iii | protein phosphorylation |
| **Nervous System Process (GO:0050877)** | | |
| AAEL000075 | gustatory receptor Gr9 | taste receptor activity |
| AAEL004006 | acetylcholine receptor protein alpha 1, 2, 3, 4 invertebrates | acetylcholine-gated cation-selective channel activity;extracellular ligand-gated ion channel activity;ion channel activity;transmembrane signaling receptor activity |
| AAEL005621 | long wavelength sensitive opsin | G protein-coupled receptor activity;photoreceptor activity |
| AAEL005625 | long wavelength sensitive opsin | G protein-coupled receptor activity;photoreceptor activity |
| AAEL005680 | Odorant receptor [Source:UniProtKB/TrEMBL;Acc:Q179B4] | odorant binding;olfactory receptor activity |
| AAEL006259 | long wavelength sensitive opsin | G protein-coupled receptor activity;photoreceptor activity |
| AAEL006685 | G-protein, gamma-subunit, putative | GTPase activity |
| AAEL009991 | myosin iii | ATP binding;actin binding;cytoskeletal motor activity;nucleotide binding;protein binding;protein kinase activity |
| AAEL010416 | unspecified product | N/A |
| AAEL010570 | acetylcholine receptor, beta-type subunit invertebrate | acetylcholine-gated cation-selective channel activity;extracellular ligand-gated ion channel activity;ion channel activity;transmembrane signaling receptor activity |
| AAEL011174 | gustatory receptor Gr11 | taste receptor activity |
| **G protein-coupled receptor signaling pathway (GO:0007186)** | | |
| AAEL000229 | prosialokinin precursor | neuropeptide signaling pathway |
| AAEL001724 | GPCR Orphan/Putative Class B Family | G protein-coupled receptor signaling pathway |
| AAEL002055 | neuroendocrine protein 7b2 | neuropeptide signaling pathway |
| AAEL005252 | corazonin | neuropeptide signaling pathway; positive regulation of heart contraction |
| AAEL005444 | pyrokinin, putative | neuropeptide signaling pathway |
| AAEL005621 | long wavelength sensitive opsin | G protein-coupled receptor signaling pathway;phototransduction;protein-chromophore linkage;response to stimulus;signal transduction;visual perception |
| AAEL005625 | long wavelength sensitive opsin | G protein-coupled receptor signaling pathway;phototransduction;protein-chromophore linkage;response to stimulus;signal transduction;visual perception |
| AAEL006232 | GPCR Orphan/Putative Class D Family | G protein-coupled receptor signaling pathway;cell surface receptor signaling pathway |
| AAEL006259 | long wavelength sensitive opsin | G protein-coupled receptor signaling pathway;phototransduction;protein-chromophore linkage;response to stimulus;signal transduction;visual perception |
| AAEL006636 | GPCR Leukokinin Family | G protein-coupled receptor signaling pathway;neuropeptide signaling pathway;signal transduction |
| AAEL006685 | G-protein, gamma-subunit, putative | G protein-coupled receptor signaling pathway;phospholipase C-activating G protein-coupled receptor signaling pathway;sensory perception of taste;signal transduction |
| AAEL006685 | G-protein, gamma-subunit, putative | N/A |
| AAEL006947 | GPCR Neurokinin/Tachykinin Family | G protein-coupled receptor signaling pathway;signal transduction;tachykinin receptor signaling pathway |
| AAEL007293 | cAMP-dependent protein kinase catalytic subunit | G protein-coupled receptor signaling pathway;phosphorylation;protein phosphorylation |
| AAEL010506 | GTP-binding protein alpha subunit, gna | G protein-coupled receptor signaling pathway;signal transduction |
| AAEL011325 | gonadotropin-releasing hormone receptor | G protein-coupled receptor signaling pathway;signal transduction |
| AAEL012887 | unspecified product | G protein-coupled receptor signaling pathway;cell surface receptor signaling pathway |
| AAEL017181 | GPCR Muscarinic Acetylcholine Family | G protein-coupled acetylcholine receptor signaling pathway;G protein-coupled receptor signaling pathway;signal transduction |
| AAEL019445 | unspecified product | G protein-coupled receptor signaling pathway |
| AAEL019691 | Short neuropeptide F [Source:UniProtKB/Swiss-Prot;Acc:A0SIX6] | neuropeptide signaling pathway |
| AAEL019804 | unspecified product | G protein-coupled receptor signaling pathway |
| AAEL019805 | unspecified product | G protein-coupled receptor signaling pathway |
| AAEL019881 | Sex peptide receptor [Source:UniProtKB/TrEMBL;Acc:B0F4E9] | G protein-coupled receptor signaling pathway |
| AAEL021016 | unspecified product | cell surface receptor signaling pathway |
| AAEL024630 | unspecified product | neuropeptide signaling pathway |
| AAEL027918 | unspecified product | G protein-coupled receptor signaling pathway;response to pheromone |
| **Odorant binding (GO:0005549)** | | |
| AAEL002587 | odorant binding protein OBP11 | N/A |
| AAEL002591 | odorant binding protein OBP13 | N/A |
| AAEL002617 | odorant binding protein OBP12 | N/A |
| AAEL002726 | D7 protein, putative | N/A |
| AAEL004729 | unspecified product | N/A |
| AAEL004730 | unspecified product | N/A |
| AAEL005680 | Odorant receptor [Source:UniProtKB/TrEMBL;Acc:Q179B4] | detection of chemical stimulus involved in sensory perception of smell;response to stimulus;sensory perception of smell;signal transduction |
| AAEL005772 | odorant binding protein OBP22 | N/A |
| AAEL006406 | Putative 14.5 kDa secreted protein [Source:UniProtKB/TrEMBL;Acc:Q8T9T4] | N/A |
| AAEL006417 | D7 protein, putative | N/A |
| AAEL006423 | Short form D7Cclu23 salivary protein [Source:UniProtKB/TrEMBL;Acc:Q95V89] | N/A |
| AAEL008013 | odorant binding protein OBP38 | N/A |
| AAEL008620 | D7 protein, putative | N/A |
| AAEL018025 | unspecified product | N/A |
| AAEL018102 | unspecified product | N/A |
| AAEL019475 | unspecified product | chromatin remodeling;histone H3-K79 methylation;histone exchange;regulation of cell cycle |
| AAEL024303 | unspecified product | N/A |
| **Innate immune response-activating signaling pathway (GO:0002758)** | | |
| AAEL007619 | Toll-like receptor | signal transduction |
| AAEL007768 | TOLL pathway signalling. | MyD88-dependent toll-like receptor signaling pathway;positive regulation of I-kappaB kinase/NF-kappaB signaling;signal transductionsignal transduction |
| AAEL013441 | Toll-like receptor | signal transduction |
| AAEL014896 | unspecified product | signal transduction |
| AAEL025498 | unspecified product | negative regulation of MyD88-independent toll-like receptor signaling pathway;positive regulation of signal transduction;response to axon injury;signal transduction |
| **Innate immune response (GO:0045087)** | | |
| AAEL007619 | Toll-like receptor | signal transduction |
| AAEL007626 | Gram-Negative Binding Protein (GNBP)  or Beta-1 3-Glucan Binding Protein (BGBP). | carbohydrate metabolic process |
| AAEL007768 | TOLL pathway signalling. | MyD88-dependent toll-like receptor signaling pathway;positive regulation of I-kappaB kinase/NF-kappaB signaling;signal transduction |
| AAEL010171 | peptidoglycan recognition protein (Long) | immune system process;innate immune response;peptidoglycan catabolic process |
| AAEL011500 | Cdc42 protein, putative | N/A |
| AAEL013139 | GTPase_rho | small GTPase mediated signal transduction |
| AAEL013441 | Toll-like receptor | signal transduction |
| AAEL014640 | Peptidoglycan Recognition Protein (Long) | peptidoglycan catabolic process |
| AAEL014896 | unspecified product | signal transduction |
| AAEL019745 | unspecified product | peptidoglycan catabolic process |
| AAEL025498 | unspecified product | negative regulation of MyD88-independent toll-like receptor signaling pathway;positive regulation of signal transduction;response to axon injury;signal transduction |
| AAEL029104 | unspecified product | antibacterial humoral response;innate immune response |
| **Immune response (GO:0006955)** | | |
| AAEL007619 | Toll-like receptor | signal transduction |
| AAEL007626 | Gram-Negative Binding Protein (GNBP)  or  Beta-1 3-Glucan Binding Protein (BGBP). | carbohydrate metabolic process |
| AAEL007768 | TOLL pathway signalling. | MyD88-dependent toll-like receptor signaling pathway;positive regulation of I-kappaB kinase/NF-kappaB signaling;signal transduction |
| AAEL010171 | peptidoglycan recognition protein (Long) | immune system process;innate immune response;peptidoglycan catabolic process |
| AAEL010524 | unspecified product | immune response;signal transduction |
| AAEL011500 | Cdc42 protein, putative | N/A |
| AAEL013139 | GTPase_rho | small GTPase mediated signal transduction |
| AAEL013441 | Toll-like receptor | signal transduction |
| AAEL014640 | Peptidoglycan Recognition Protein (Long) | peptidoglycan catabolic process |
| AAEL014896 | unspecified product | signal transduction |
| AAEL014990 | unspecified product | N/A |
| AAEL019745 | unspecified product | peptidoglycan catabolic process |
| AAEL025498 | unspecified product | negative regulation of MyD88-independent toll-like receptor signaling pathway;positive regulation of signal transduction;response to axon injury;signal transduction |
| AAEL029104 | unspecified product | antibacterial humoral response;innate immune response |

Table S4: Differentially expressed genes GO enriched at four different time points in a LD cycle, specifically for GO:BP, Sensory perception (GO:0007600), Nervous System Process (GO:0050877), G protein-coupled receptor signaling pathway (GO:0007186), Odorant binding (GO:0005549), Immune response (GO:0006955), etc.

| **Gene Id** | **Product Description** | **Computed GO Processes** |
| --- | --- | --- |
| **Sensory perception (GO:0007600)** | | |
| AAEL000075 | gustatory receptor Gr9 | taste receptor activity |
| AAEL005621 | long wavelength sensitive opsin | G protein-coupled receptor activity;photoreceptor activity |
| AAEL005625 | long wavelength sensitive opsin | G protein-coupled receptor activity;photoreceptor activity |
| AAEL005680 | Odorant receptor [Source:UniProtKB/TrEMBL;Acc:Q179B4] | odorant binding;olfactory receptor activity |
| AAEL006259 | long wavelength sensitive opsin | G protein-coupled receptor activity;photoreceptor activity |
| AAEL006685 | G-protein, gamma-subunit, putative | GTPase activity |
| AAEL009991 | myosin iii | N/A |
| AAEL010416 | unspecified product | ATP binding; actin binding;cytoskeletal motor activity;nucleotide binding;protein binding;protein kinase activity |
| AAEL011174 | gustatory receptor Gr11 | taste receptor activity |
| AAEL005621 | long wavelength sensitive opsin | G protein-coupled receptor signaling pathway;phototransduction;protein-chromophore linkage;response to stimulus;signal transduction;visual perception |
| AAEL005625 | long wavelength sensitive opsin | G protein-coupled receptor signaling pathway;phototransduction;protein-chromophore linkage;response to stimulus;signal transduction;visual perception |
| AAEL006259 | long wavelength sensitive opsin | G protein-coupled receptor signaling pathway;phototransduction;protein-chromophore linkage;response to stimulus;signal transduction;visual perception |
| AAEL009991 | myosin iii | protein phosphorylation |
| **Nervous System Process (GO:0050877)** | | |
| AAEL000075 | gustatory receptor Gr9 | taste receptor activity |
| AAEL004006 | acetylcholine receptor protein alpha 1, 2, 3, 4 invertebrates | acetylcholine-gated cation-selective channel activity;extracellular ligand-gated ion channel activity;ion channel activity;transmembrane signaling receptor activity |
| AAEL005621 | long wavelength sensitive opsin | G protein-coupled receptor activity;photoreceptor activity |
| AAEL005625 | long wavelength sensitive opsin | G protein-coupled receptor activity;photoreceptor activity |
| AAEL005680 | Odorant receptor [Source:UniProtKB/TrEMBL;Acc:Q179B4] | odorant binding;olfactory receptor activity |
| AAEL006259 | long wavelength sensitive opsin | G protein-coupled receptor activity;photoreceptor activity |
| AAEL006685 | G-protein, gamma-subunit, putative | GTPase activity |
| AAEL009991 | myosin iii | ATP binding;actin binding;cytoskeletal motor activity;nucleotide binding;protein binding;protein kinase activity |
| AAEL010416 | unspecified product | N/A |
| AAEL010570 | acetylcholine receptor, beta-type subunit invertebrate | acetylcholine-gated cation-selective channel activity;extracellular ligand-gated ion channel activity;ion channel activity;transmembrane signaling receptor activity |
| AAEL011174 | gustatory receptor Gr11 | taste receptor activity |
| **G protein-coupled receptor signaling pathway (GO:0007186)** | | |
| AAEL000229 | prosialokinin precursor | neuropeptide signaling pathway |
| AAEL001724 | GPCR Orphan/Putative Class B Family | G protein-coupled receptor signaling pathway |
| AAEL002055 | neuroendocrine protein 7b2 | neuropeptide signaling pathway |
| AAEL005252 | corazonin | neuropeptide signaling pathway; positive regulation of heart contraction |
| AAEL005444 | pyrokinin, putative | neuropeptide signaling pathway |
| AAEL005621 | long wavelength sensitive opsin | G protein-coupled receptor signaling pathway;phototransduction;protein-chromophore linkage;response to stimulus;signal transduction;visual perception |
| AAEL005625 | long wavelength sensitive opsin | G protein-coupled receptor signaling pathway;phototransduction;protein-chromophore linkage;response to stimulus;signal transduction;visual perception |
| AAEL006232 | GPCR Orphan/Putative Class D Family | G protein-coupled receptor signaling pathway;cell surface receptor signaling pathway |
| AAEL006259 | long wavelength sensitive opsin | G protein-coupled receptor signaling pathway;phototransduction;protein-chromophore linkage;response to stimulus;signal transduction;visual perception |
| AAEL006636 | GPCR Leukokinin Family | G protein-coupled receptor signaling pathway;neuropeptide signaling pathway;signal transduction |
| AAEL006685 | G-protein, gamma-subunit, putative | G protein-coupled receptor signaling pathway;phospholipase C-activating G protein-coupled receptor signaling pathway;sensory perception of taste;signal transduction |
| AAEL006685 | G-protein, gamma-subunit, putative | N/A |
| AAEL006947 | GPCR Neurokinin/Tachykinin Family | G protein-coupled receptor signaling pathway;signal transduction;tachykinin receptor signaling pathway |
| AAEL007293 | cAMP-dependent protein kinase catalytic subunit | G protein-coupled receptor signaling pathway;phosphorylation;protein phosphorylation |
| AAEL010506 | GTP-binding protein alpha subunit, gna | G protein-coupled receptor signaling pathway;signal transduction |
| AAEL011325 | gonadotropin-releasing hormone receptor | G protein-coupled receptor signaling pathway;signal transduction |
| AAEL012887 | unspecified product | G protein-coupled receptor signaling pathway;cell surface receptor signaling pathway |
| AAEL017181 | GPCR Muscarinic Acetylcholine Family | G protein-coupled acetylcholine receptor signaling pathway;G protein-coupled receptor signaling pathway;signal transduction |
| AAEL019445 | unspecified product | G protein-coupled receptor signaling pathway |
| AAEL019691 | Short neuropeptide F [Source:UniProtKB/Swiss-Prot;Acc:A0SIX6] | neuropeptide signaling pathway |
| AAEL019804 | unspecified product | G protein-coupled receptor signaling pathway |
| AAEL019805 | unspecified product | G protein-coupled receptor signaling pathway |
| AAEL019881 | Sex peptide receptor [Source:UniProtKB/TrEMBL;Acc:B0F4E9] | G protein-coupled receptor signaling pathway |
| AAEL021016 | unspecified product | cell surface receptor signaling pathway |
| AAEL024630 | unspecified product | neuropeptide signaling pathway |
| AAEL027918 | unspecified product | G protein-coupled receptor signaling pathway;response to pheromone |
| **Odorant binding (GO:0005549)** | | |
| AAEL002587 | odorant binding protein OBP11 | N/A |
| AAEL002591 | odorant binding protein OBP13 | N/A |
| AAEL002617 | odorant binding protein OBP12 | N/A |
| AAEL002726 | D7 protein, putative | N/A |
| AAEL004729 | unspecified product | N/A |
| AAEL004730 | unspecified product | N/A |
| AAEL005680 | Odorant receptor [Source:UniProtKB/TrEMBL;Acc:Q179B4] | detection of chemical stimulus involved in sensory perception of smell;response to stimulus;sensory perception of smell;signal transduction |
| AAEL005772 | odorant binding protein OBP22 | N/A |
| AAEL006406 | Putative 14.5 kDa secreted protein [Source:UniProtKB/TrEMBL;Acc:Q8T9T4] | N/A |
| AAEL006417 | D7 protein, putative | N/A |
| AAEL006423 | Short form D7Cclu23 salivary protein [Source:UniProtKB/TrEMBL;Acc:Q95V89] | N/A |
| AAEL008013 | odorant binding protein OBP38 | N/A |
| AAEL008620 | D7 protein, putative | N/A |
| AAEL018025 | unspecified product | N/A |
| AAEL018102 | unspecified product | N/A |
| AAEL019475 | unspecified product | chromatin remodeling;histone H3-K79 methylation;histone exchange;regulation of cell cycle |
| AAEL024303 | unspecified product | N/A |
| **Innate immune response-activating signaling pathway (GO:0002758)** | | |
| AAEL007619 | Toll-like receptor | signal transduction |
| AAEL007768 | TOLL pathway signalling. | MyD88-dependent toll-like receptor signaling pathway;positive regulation of I-kappaB kinase/NF-kappaB signaling;signal transductionsignal transduction |
| AAEL013441 | Toll-like receptor | signal transduction |
| AAEL014896 | unspecified product | signal transduction |
| AAEL025498 | unspecified product | negative regulation of MyD88-independent toll-like receptor signaling pathway;positive regulation of signal transduction;response to axon injury;signal transduction |
| **Innate immune response (GO:0045087)** | | |
| AAEL007619 | Toll-like receptor | signal transduction |
| AAEL007626 | Gram-Negative Binding Protein (GNBP)  or Beta-1 3-Glucan Binding Protein (BGBP). | carbohydrate metabolic process |
| AAEL007768 | TOLL pathway signalling. | MyD88-dependent toll-like receptor signaling pathway;positive regulation of I-kappaB kinase/NF-kappaB signaling;signal transduction |
| AAEL010171 | peptidoglycan recognition protein (Long) | immune system process;innate immune response;peptidoglycan catabolic process |
| AAEL011500 | Cdc42 protein, putative | N/A |
| AAEL013139 | GTPase_rho | small GTPase mediated signal transduction |
| AAEL013441 | Toll-like receptor | signal transduction |
| AAEL014640 | Peptidoglycan Recognition Protein (Long) | peptidoglycan catabolic process |
| AAEL014896 | unspecified product | signal transduction |
| AAEL019745 | unspecified product | peptidoglycan catabolic process |
| AAEL025498 | unspecified product | negative regulation of MyD88-independent toll-like receptor signaling pathway;positive regulation of signal transduction;response to axon injury;signal transduction |
| AAEL029104 | unspecified product | antibacterial humoral response;innate immune response |
| **Immune response (GO:0006955)** | | |
| AAEL007619 | Toll-like receptor | signal transduction |
| AAEL007626 | Gram-Negative Binding Protein (GNBP)  or  Beta-1 3-Glucan Binding Protein (BGBP). | carbohydrate metabolic process |
| AAEL007768 | TOLL pathway signalling. | MyD88-dependent toll-like receptor signaling pathway;positive regulation of I-kappaB kinase/NF-kappaB signaling;signal transduction |
| AAEL010171 | peptidoglycan recognition protein (Long) | immune system process;innate immune response;peptidoglycan catabolic process |
| AAEL010524 | unspecified product | immune response;signal transduction |
| AAEL011500 | Cdc42 protein, putative | N/A |
| AAEL013139 | GTPase_rho | small GTPase mediated signal transduction |
| AAEL013441 | Toll-like receptor | signal transduction |
| AAEL014640 | Peptidoglycan Recognition Protein (Long) | peptidoglycan catabolic process |
| AAEL014896 | unspecified product | signal transduction |
| AAEL014990 | unspecified product | N/A |
| AAEL019745 | unspecified product | peptidoglycan catabolic process |
| AAEL025498 | unspecified product | negative regulation of MyD88-independent toll-like receptor signaling pathway;positive regulation of signal transduction;response to axon injury;signal transduction |
| AAEL029104 | unspecified product | antibacterial humoral response;innate immune response |


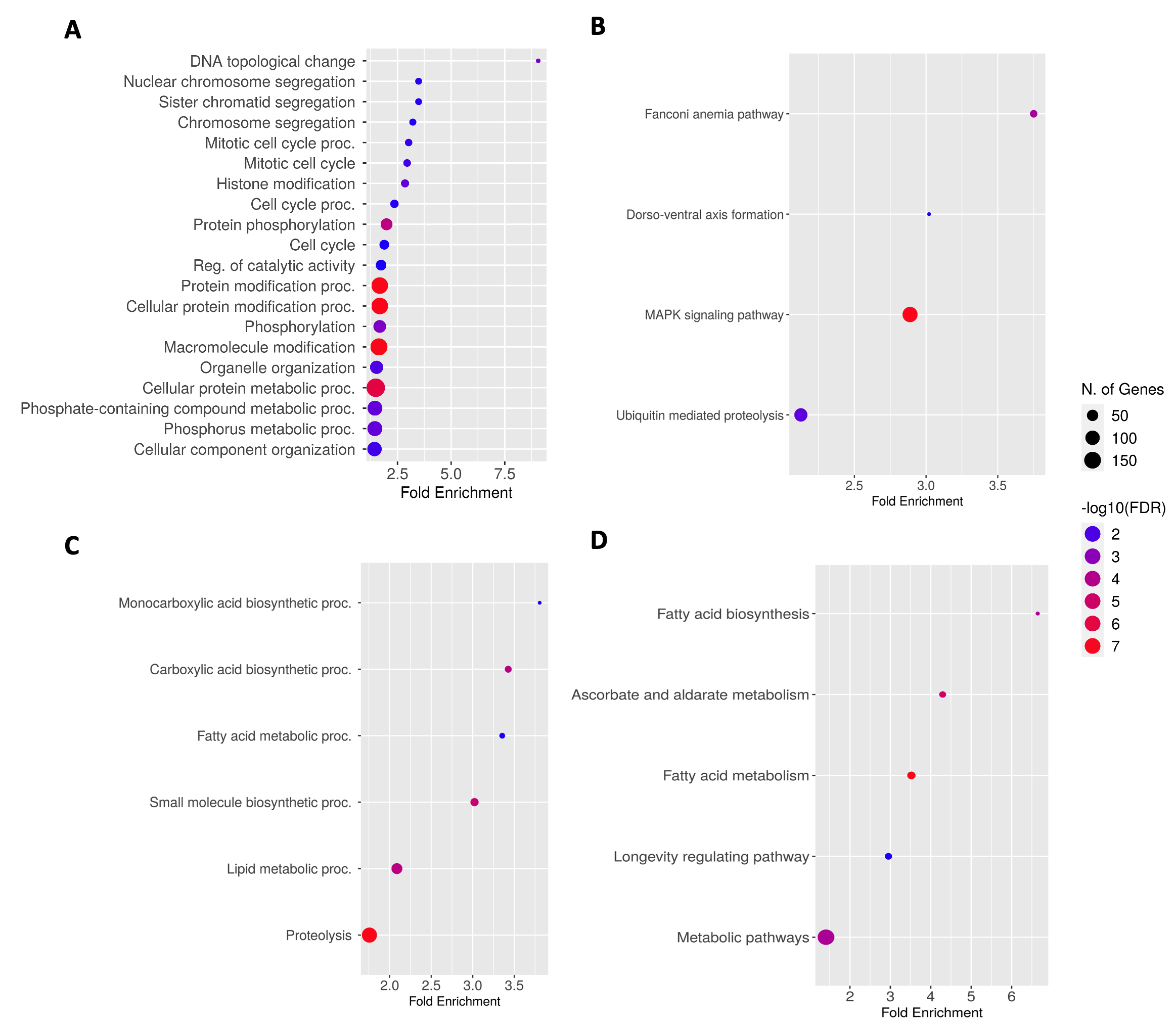


Figure S1: Gene ontology (GO) enrichment analysis for rhythmically differentially expressed genes in *Cyc* KO-specific and WT-specific groups. (A) Enriched GO term biological processes for *Cyc* KO-specific genes. (B) Enriched KEGG pathways for *Cyc* KO-specific genes. (C) Enriched GO term biological processes for WT specific genes. (D) Enriched KEGG pathways for WT specific genes. Only the top 20 were shown. The *x*-axis represents the proportion of genes that belong to a given functional category to the total number of differentially expressed genes. *p*-values were corrected using the Benjamini–Hochberg method.
